# MicroRNAs and Cellular Senescence in Melanoma: An Underexplored Link to Tumor Progression—A Systematic Review with Bioinformatics Analyses

**DOI:** 10.3390/ijms27146462

**Published:** 2026-07-21

**Authors:** Sabina Beganović, Tainara Marcansoni, Virginia Lazzari, José Eduardo Vargas

**Affiliations:** 1 Department of Cell Biology, Federal University of Paraná (UFPR), Curitiba 80060-000, PR, Brazil; sabinabeganovic@ufpr.br (S.B.); tainaramarcansoni@ufpr.br (T.M.); 2 Department of Cell Biology, Embryology and Genetics, Center for Biological Sciences, Federal University of Santa Catarina (UFSC), Florianópolis 88040-900, SC, Brazil; virginia.lazzari@ufsc.br

**Keywords:** skin cancer, cell cycle, cutaneous melanoma, non-coding RNAs, systematic review, bioinformatic analysis

## Abstract

MicroRNAs are important regulators of melanoma progression; however, their relationship with cellular senescence remains poorly understood. To address this gap, a systematic review was conducted following PRISMA 2020 guidelines and prospectively registered in the International Prospective Register of Systematic Reviews (PROSPERO) under registration number CRD420251155760. A comprehensive search of PubMed, Scopus, Embase, and Dimensions identified studies evaluating melanoma-associated microRNAs and their effects on cell cycle regulation. Risk of bias was assessed using the SYRCLE tool and an adapted version of ToxRTool, with the included studies classified as having low, moderate, or high risk of bias. Fifteen studies met the eligibility criteria. Most studies reported that microRNA modulation reduced melanoma proliferation through cell cycle arrest; however, only two directly assessed senescence-associated markers. Of the fifteen identified microRNAs, seven had predicted targets and were included in the bioinformatic analysis. Integration of these predictions with genes downregulated in high-risk melanoma and underexpressed during cellular senescence identified 158 shared genes. Subsequent analysis identified predicted targets within this gene set only for hsa-miR-195-5p, and hsa-miR-425-5p, highlighting *RNF138*, and *SYNCRIP* as candidate regulatory genes. Collectively, these findings suggest a potential link between microRNA-mediated regulation and senescence-associated pathways during melanoma progression.

## 1. Introduction

Cutaneous melanoma is an aggressive form of skin cancer characterized by high metastatic potential and poor clinical prognosis [1,2]. Although less prevalent than other cutaneous malignancies, melanoma accounts for most skin cancer-related deaths, being responsible for more than 90% of mortality associated with these neoplasms [3]. Current epidemiological data indicates a continuous and substantial increase in the global incidence of melanoma, reinforcing the need to identify novel molecular targets, develop innovative therapeutic strategies, and establish reliable biomarkers associated with tumor progression, prognosis, and disease aggressiveness [3,4].

Approximately a decade ago, therapeutic options for melanoma were largely restricted to conventional cytotoxic chemotherapy, which showed limited efficacy and was associated with high rates of recurrence and metastasis [5]. Agents such as dacarbazine and temozolomide produced only modest clinical responses [6,7,8,9], while the subsequent incorporation of immunotherapy significantly improved patient outcomes, although resistance and disease progression remain major clinical challenges [10,11].

Alternative therapeutic strategies aimed at controlling melanoma progression have gained increasing attention, particularly those targeting cell cycle regulation and the induction of cellular senescence (CS). This process is characterized by irreversible cell cycle arrest accompanied by metabolic, morphological, and transcriptional alterations [12,13]. Therapy-induced senescence, beyond its classical role as a tumor-suppressive mechanism, has emerged as an important component of melanoma treatment responses and represents a promising therapeutic window in the context of chemotherapy, molecular targeted therapies, and immunotherapeutic approaches [13,14,15,16]. However, the current limitations of these treatments are partially associated with the marked heterogeneity of the melanoma tumor microenvironment, which evolves from a relatively organized structure in early-stage disease to a highly complex and immunosuppressive landscape during tumor progression [10,17,18].

Tumor microenvironment and persistence of senescent cells may also contribute to tumor maintenance and disease progression through the senescence-associated secretory phenotype (SASP), a complex secretory phenotype characterized by the release of inflammatory cytokines, chemokines, growth factors, metalloproteinases, and extracellular vesicles (EVs) [12,14,17]. These factors contribute to a pro-tumorigenic microenvironment associated with therapeutic resistance, tumor invasion, angiogenesis, immune modulation, and sustained melanoma cell proliferation [15,17,19]. This dual role of senescence, acting as both a tumor-suppressive and tumor-promoting process, highlights the biological complexity of melanoma progression and therapeutic response.

Among the components of the SASP, EVs have emerged as particularly relevant mediators of intercellular communication during melanoma progression [20,21]. EVs released by senescent melanoma and stromal cells transport diverse bioactive cargoes, including proteins, lipids, DNA fragments, long non-coding RNAs (lncRNAs), and microRNAs, thereby influencing pathways associated with cell cycle progression [22], immune evasion [23,24], therapeutic resistance [25,26] and metastatic dissemination [20,27].

MicroRNAs stand out as key post-transcriptional regulators capable of controlling the expression of genes involved in proliferation [28,29,30], apoptosis [31,32], DNA damage responses [33,34], and senescence-associated pathways [35,36]. Accumulating evidence has demonstrated that specific microRNAs may suppress melanoma progression by inducing cell cycle arrest, promoting senescence-associated mechanisms, and sensitizing tumor cells to conventional and targeted therapies [36,37,38]. Furthermore, the dysregulated expression of microRNAs, both intracellularly and extracellularly through EV-associated transport, has been increasingly linked to tumor aggressiveness, therapeutic response, and patient prognosis [39,40].

Despite growing evidence supporting the role of microRNAs in melanoma biology, the literature remains fragmented regarding their involvement in the regulation of CS and cell cycle dynamics. Understanding the contribution of these molecules to such processes has become increasingly relevant given their capacity to modulate pathways associated with both tumor suppression and tumor progression. Moreover, no systematic review has specifically synthesized the available evidence linking microRNAs, senescence, and cell cycle regulation in melanoma. Therefore, this research aimed to comprehensively evaluate the role of microRNAs in the regulation of CS and cell cycle progression in cutaneous melanoma and to examine how these molecular mechanisms may contribute to tumor progression through a systematic review followed by a bioinformatics analysis.

## 2. Materials and Methods

A systematic review of the literature was conducted in accordance with the Preferred Reporting Items for Systematic Reviews and Meta-Analyses (PRISMA 2020) guidelines, with the aim of analyzing the role of microRNAs in regulating the cell cycle and their potential influence on senescent cells in cutaneous melanoma. The review protocol was prospectively registered in PROSPERO under registration number CRD420251155760. The registration is publicly available at: https://www.crd.york.ac.uk/PROSPERO/view/CRD420251155760, accessed on 16 May 2026.

### 2.1. Eligibility Criteria

We included studies that evaluated the activity of microRNAs in the context of cutaneous melanoma, particularly those related to cell cycle regulation and CS processes. All experimental designs that included cell cycle arrest analyses, senescence assays, and microRNA quantification were considered eligible. Outcomes of interest included the assessment of the cell cycle phases in which the cells were located, as well as the identification of potential senescent cells.

To formulate the research question, the PICO strategy was used, in which the population (P) consisted of melanoma cells; the intervention (I) involved microRNA activity, while studies investigating extracellular vesicle-mediated RNA communication were also considered eligible when they evaluated RNA-mediated mechanisms affecting melanoma cell cycle regulation or proliferation; the comparison (C) included control or untreated cells; and the outcome (O) was the induction of cell cycle arrest or CS.

Systematic and narrative reviews, case reports, and editorials were excluded, as were studies not published in English, with no restriction on publication date. Studies conducted in humans and mice were included, whereas studies involving any other populations were excluded. Studies involving melanoma types other than cutaneous melanoma were also excluded. Subsequently, during the full-text review, studies that did not evaluate CS or cell cycle regulation through functional assays or molecular markers associated with cell cycle arrest were excluded.

### 2.2. Information Sources

The literature search was conducted by reviewer S.B. on 25 November 2025, in PubMed, Scopus, Embase, and Dimensions databases, with no date restrictions applied.

### 2.3. Search Strategy and Selection Process

A standardized search strategy was developed using predefined keywords and boolean operators to ensure consistency across all databases. The search terms were: (“melanoma” OR “melanoma cells”) AND (“microRNA” OR “miRNA”) AND (“tumor progression” OR “tumor growth” OR “cancer progression”). Terms directly related to CS were intentionally not included in the search strategy to avoid excluding melanoma–microRNA studies investigating cell-cycle regulation without explicitly describing the observed phenotype as senescent.

Database-specific search strings, including any necessary adaptations and applied filters, are provided in Table 1.

Records retrieved from each database were imported into the Rayyan platform by one reviewer (V.L.), enabling two additional reviewers (S.B. and J.E.V.) to perform a blinded screening process. Duplicate records were automatically identified by the software and subsequently verified by one reviewer (J.E.V.) before removal. Two reviewers (S.B. and J.E.V.) then independently screened the titles and abstracts of the remaining studies according to the predefined inclusion and exclusion criteria. Subsequently, the full texts of potentially eligible articles were independently assessed by S.B., J.E.V., and T.M. to confirm their eligibility for inclusion in the systematic review. Any disagreements were resolved through discussion until consensus was reached.

### 2.4. Data Extraction

Extracted data included authors, year of publication, article title, journal of publication and journal impact factor, country and primary institution where the research was conducted, experimental design, sample size, population, intervention or exposure, assessment methods, biological markers, variables analyzed, results and conclusions of the experiments, as well as the presence or absence of conflicts of interest. Additionally, reviewer S.B. conducted a critical analysis of the strengths and limitations of each included study. The complete information is organized in the Supplementary Material (Table S1). Due to the methodological heterogeneity observed among the included studies, it was not possible to perform a meta-analysis. Thus, the data were analyzed descriptively and qualitatively.

### 2.5. Study Risk of Bias Assessment

The risk of bias assessment for the included studies was performed according to the experimental design adopted in each article. For in vivo studies, the SYRCLE Risk of Bias Tool was used, evaluating domains related to randomization, allocation concealment, baseline comparability between groups, blinding of interventions and outcome assessment, incomplete outcome data, and the adequacy of experimental conditions.

The methodological quality of the included in vitro studies was evaluated using an adapted version of the Toxicological Data Reliability Assessment Tool (ToxRTool) described by Schneider et al. [41]. Studies were assessed for experimental control, replication, method standardization, cell line authentication, contamination testing, experimental conditions, blinding (when applicable), and statistical analysis. Each item was scored as fulfilled (1), not fulfilled (0), or not applicable (N/A). Risk of bias was independently assessed by two reviewers (S.B. and T.M.), and any disagreements were resolved through consultation with a third reviewer (J.E.V.) until consensus was reached.

The final score for each study was calculated as the ratio between the obtained score and the maximum score applicable to its respective experimental model, acknowledging that some studies included both in vivo and in vitro experiments, whereas others employed only one model. Studies with scores ranging from 0.00 to 0.30 were classified as having a high risk of bias, those scoring from 0.31 to 0.60 as having a moderate risk of bias, and those scoring from 0.61 to 1.00 as having a low risk of bias. The classification thresholds were established by dividing the total score range into three categories representing high, moderate, and low risk of bias. The evaluated criteria and the corresponding scores assigned to each study are presented in Supplementary Material (Table S2).

### 2.6. Identification of Senescence-Associated Downregulated Genes and MicroRNA Target Prediction

Differentially expressed genes (DEGs) were obtained from [42] which performed a transcriptomic comparison between high-risk and low-risk skin cutaneous melanoma (SKCM). These patients were classified according to a Cellular Senescence-Related Signature (CSRS). The original study analyzed transcriptomic and clinical data from 469 SKCM patients from The Cancer Genome Atlas (TCGA-SKCM). The CSRS was developed using senescence-related genes retrieved from the CellAge database [43] and prognostic modeling based on univariate and LASSO Cox regression analyses. Patients were subsequently stratified into high- and low-risk groups according to the median CSRS score, and DEGs between these groups were identified. For the present study, only genes significantly downregulated in the high-risk group (FDR < 0.01 and log_2_FC ≤ −1) were selected. The resulting gene list was intersected with the CellAge transcriptional signature dataset (CellAge Build 3), accessed on 25 May 2026, specifically considering genes reported as underexpressed during CS.

All microRNAs identified in the studies, after the application of the inclusion and exclusion criteria in the systematic review, were subjected to target prediction using miRWalk 2.0 [44] (accessed on 28 May 2026 from http://mirwalk.umm.uni-heidelberg.de/). A stringent miRWalk score threshold (≥0.90), together with confirmation by TargetScan, miRDB, and miRTarBase, was applied to prioritize high-confidence microRNA–target interactions and minimize false-positive predictions before downstream analyses. The resulting list of predicted microRNA targets was subsequently intersected with the senescence-associated downregulated genes, identified in the previous step. The rationale of this approach was to identify senescence-associated genes that are transcriptionally suppressed in high-risk melanoma and may be post-transcriptionally regulated by melanoma-associated microRNAs, thereby revealing candidate molecular mechanisms linking CS and melanoma progression. Venn diagrams were generated using the online Venn diagram tool (accessed on 28 May 2026 from http://bioinformatics.psb.ugent.be/webtools/Venn/).

### 2.7. Protein–Protein Interaction (PPI) Network Construction and Centrality Analysis

To investigate the functional relationships among the overlapping genes identified after target prediction and intersection analyses, a protein–protein interaction (PPI) network was constructed using the STRING version 12.0 [45] with the default minimum confidence score of 0.400 (medium confidence). The default STRING confidence score was retained because it provides a balance between sensitivity and specificity for exploratory network analyses. Higher confidence thresholds generated substantially sparser interaction networks, limiting meaningful centrality analyses of the relatively small input gene set. The resulting network was imported into Cytoscape 3.10.4 [46], and topological analyses were performed using the CentiScaPe 2.2 plugin [47]. Degree and betweenness centrality parameters were calculated to identify highly connected and influential nodes within the network. Nodes presenting degree and betweenness values above the respective network averages were classified as hubs and bottlenecks, respectively, whereas nodes exhibiting both characteristics were considered hub-bottlenecks (HB). These analyses were performed to identify key senescence-associated genes and microRNA-regulated molecular interaction networks potentially involved in melanoma progression.

### 2.8. Gene Ontology Enrichment Analysis

Gene identifiers obtained from the network analysis were manually curated using the GeneCards database (https://www.genecards.org; accessed on 16 June 2026). Protein accession numbers were converted into their corresponding gene symbols through the UniProt ID mapping service (https://www.uniprot.org; accessed on 16 June 2026) [48]. The resulting curated gene set was subsequently employed in functional enrichment analyses.

**Figure 1:**
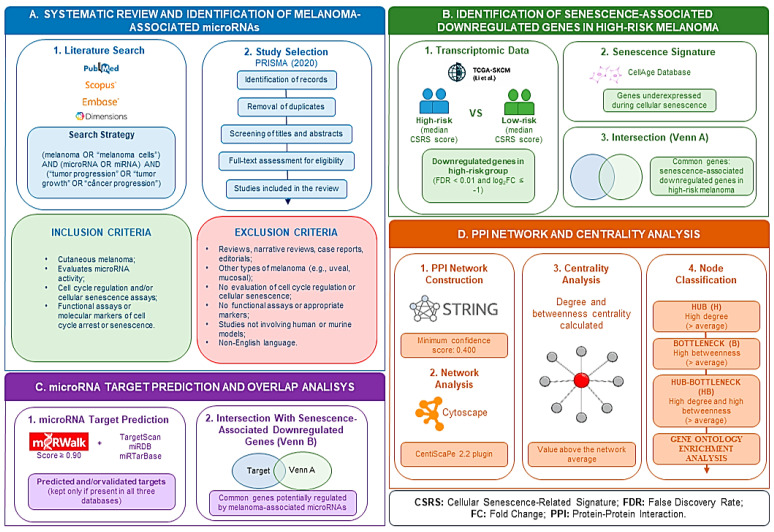
Methodological workflow for the identification and analysis of melanoma-associated microRNAs linked to CS in high-risk melanoma. (**A**) Systematic review conducted according to the PRISMA 2020 guidelines to identify melanoma-associated microRNAs involved in cell cycle regulation and/or CS. Studies were retrieved from PubMed, Scopus, Embase, and Dimensions databases and selected according to predefined inclusion and exclusion criteria. (**B**) Identification of senescence-associated genes downregulated in high-risk SKCM. DEGs downregulated in high-risk melanoma patients were obtained from [42] and intersected with genes reported as underexpressed during CS in the CellAge database (Venn A), generating a senescence-associated gene set for subsequent analyses. (**C**) Target prediction of melanoma-associated microRNAs identified in the systematic review. MicroRNA targets were predicted using miRWalk 2.0 (score ≥ 0.90) and filtered using TargetScan, miRDB, and miRTarBase databases. Predicted targets were then intersected with the senescence-associated gene set obtained in panel B (Venn B). (**D**) PPI network construction and centrality analysis of genes targeted by melanoma-associated microRNAs. The predicted target genes were analyzed using STRING v12.0 (minimum confidence score = 0.400) and Cytoscape/CentiScaPe to calculate degree and betweenness centrality metrics and classify nodes as hubs, bottlenecks, or hub-bottleneck (HB) nodes. The resulting network was subsequently subjected to functional enrichment analysis to identify overrepresented biological processes.

Functional annotation of the gene set was carried out using the BiNGO (version 3.0.5) [49] application within the Cytoscape (version 3.10.4) environment. Overrepresented Gene Ontology (GO) Biological Process categories were identified by comparing the input gene set against the selected reference annotation database. Statistical significance was determined using the Benjamini–Hochberg false discovery rate correction, and enriched terms with adjusted *p*-values below 0.001 were considered significant. A design and bioinformatics workflow is presented in Figure 1.

During the preparation of this manuscript, ChatGPT (GPT-5.5, OpenAI) was used exclusively for English grammar and language editing. No text, scientific content, data, analyses, interpretations, or conclusions were generated by artificial intelligence. All content was written, reviewed, and verified by the authors, who assumed full responsibility for the accuracy and integrity of the work.

## 3. Results

### 3.1. Search Strategy and Selection Process

**Figure 2:**
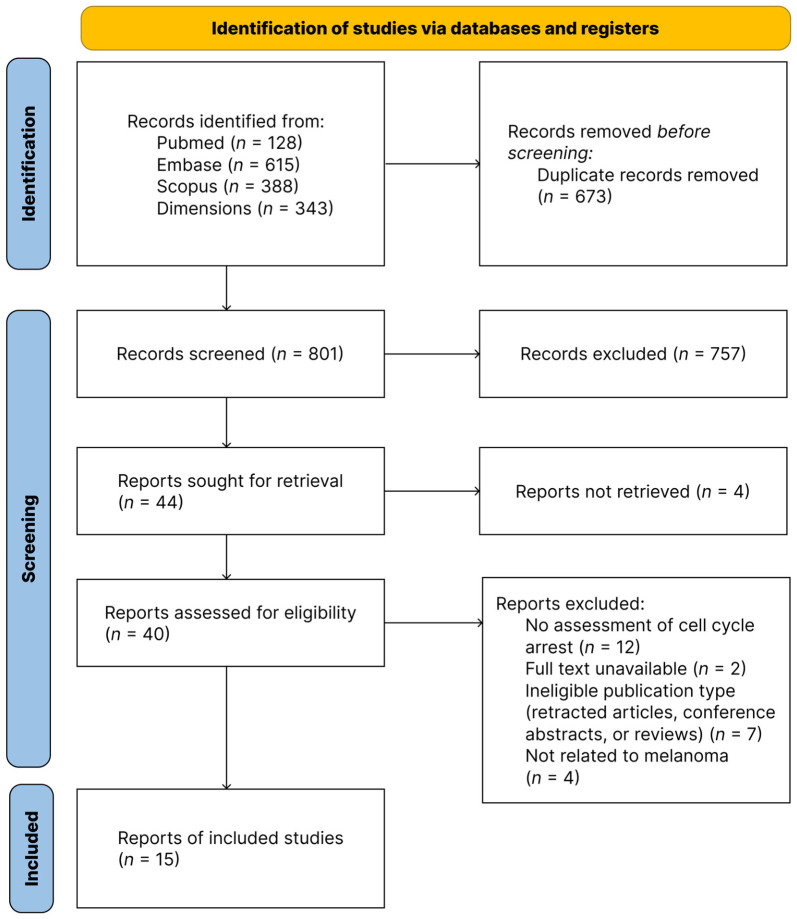
PRISMA 2020 flow diagram of the study selection process. A total of 1474 records were identified through searches conducted in PubMed (*n* = 128), Embase (*n* = 615), Scopus (*n* = 388), and Dimensions (*n* = 343). After the removal of 673 duplicate records, 801 records remained for title and abstract screening, of which 757 were excluded. Forty-four reports were sought for retrieval, and four were not retrieved. The remaining forty reports were assessed for eligibility, resulting in the exclusion of twenty-four studies due to lack of cell cycle assessment (*n* = 12), unavailable full text (*n* = 2), ineligible publication type, including retracted articles, conference abstracts, or reviews (*n* = 7), and lack of relevance to cutaneous melanoma (*n* = 4). Ultimately, fifteen studies met the eligibility criteria and were included in the systematic review.

A total of 1474 records were identified through searches conducted in the PubMed, Embase, Scopus, and Dimensions databases using specific keywords and filters (Table 1). After the removal of 673 duplicates, 801 records remained for screening. Following title and abstract screening performed independently by paired reviewers, 757 records were excluded by consensus for addressing topics unrelated to the review question or presenting inappropriate study designs, leaving 44 studies for full-text assessment. Of these, four studies were excluded after discussion between the primary reviewers and a third reviewer. Among the remaining 40 studies, 24 were excluded based on the predefined eligibility criteria: 12 for not addressing cell cycle regulation, 2 due to unavailable full text, 7 for being review articles or retracted publications, and 4 for not involving cutaneous melanoma. Ultimately, 15 studies were included in this systematic review (Figure 2).

### 3.2. Data Extraction

Data extraction was performed according to the variables presented in Table 2, including article identification, evaluated microRNAs, experimental approach, biological model, main assessments, and main biological effects. To facilitate data organization and comparative analysis, the selected studies were categorized according to their experimental approaches and biological models, encompassing in vitro, in vivo and ex vivo investigations, as well as studies combining multiple methodologies. This classification enabled a comprehensive assessment of the experimental strategies used to investigate microRNA-mediated regulation of the cell cycle in cutaneous melanoma and allowed the identification of recurring biological effects associated with tumor progression, proliferation, cell cycle arrest, and senescence-related processes. One included study [50] differed mechanistically from the remaining studies by investigating extracellular vesicle-mediated RNA communication derived from IL-33-activated eosinophils instead of direct melanoma-intrinsic microRNA manipulation, contributing to the methodological heterogeneity of the included studies. A detailed description of the experimental procedures and assays performed in each included study is provided in Supplementary Table S1.

### 3.3. Study Risk of Bias Assessment

Risk of bias was assessed according to each study’s ability to adequately address the predefined objectives of this review, considering the characteristics and limitations of each experimental approach. The evaluation was performed separately for in vitro/ex vivo and in vivo studies, based on methodological quality, experimental consistency, molecular characterization, and the relevance of the findings to cell cycle regulation, senescence, and melanoma progression. Studies with insufficient validation, limited alignment with the proposed outcomes, inadequate methodological description, or conclusions not fully supported by the data received lower scores and were classified as having a higher risk of bias (Table 3).

### 3.4. In Silico Predictions

**Figure 3:**
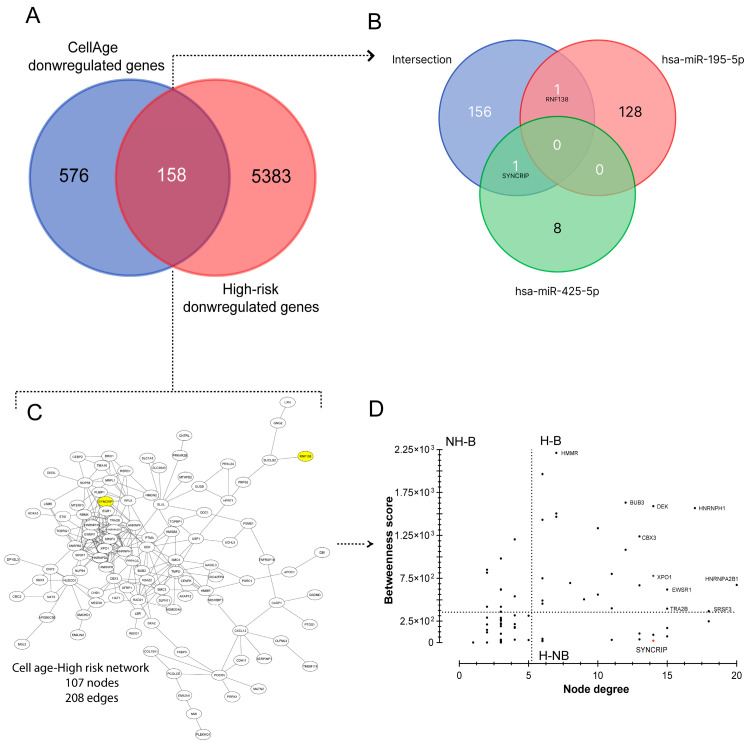
Integrative identification of senescence-associated downregulated genes potentially regulated by melanoma-associated microRNAs in high-risk melanoma. (**A**) Venn diagram showing the intersection between genes downregulated in the high-risk melanoma group and genes reported as underexpressed during CS in the CellAge dataset. (**B**) Venn diagram shows the overlap between the senescence-associated downregulated genes identified in panel A and predicted targets of melanoma-associated microRNAs identified through the systematic review. (**C**) PPI network constructed from the overlapping senescence-associated downregulated genes in high-risk melanoma. Nodes highlighted in yellow represent the predicted targets of the microRNAs. (**D**) Centrality analysis of the PPI network showing the classification of microRNA-predicted target genes as hubs, bottlenecks, or HB nodes according to degree and betweenness centrality values above the network averages. The hub node predicted to be targeted by miR-425-5p in the present study is highlighted in red. This integrative workflow aimed to identify key senescence-associated genes that are transcriptionally suppressed in high-risk melanoma and potentially modulated by melanoma-associated microRNAs.

A total of 5541 genes were identified as significantly downregulated in high-risk melanoma, whereas the CellAge database contained 734 genes reported as underexpressed during CS. The intersection of these datasets revealed 158 common genes (Figure 3A).

Target prediction analysis was subsequently performed using the fifteen melanoma-associated microRNAs identified through the systematic review. Notably, the two microRNAs previously demonstrated to regulate cellular senescence in melanoma, hsa-miR-125b and hsa-miR-344d-3-5p, were intentionally excluded from the computational target prediction analysis because the aim of this step was to identify novel senescence-associated candidate microRNAs rather than reanalyze experimentally validated interactions. Of the remaining microRNAs, only seven presented predicted target genes in the miRWalk database. Subsequent intersection analysis with the set of 158 senescence-associated downregulated genes revealed that only two microRNAs showed predicted targets related to cellular senescence. Specifically, *RNF138* was predicted as a target of hsa-miR-195-5p, and *SYNCRIP* as a target of hsa-miR-425-5p (Figure 3B). No shared targets were identified among the remaining five microRNAs, indicating a limited overlap between the predicted microRNA regulatory network and senescence-associated genes suppressed in high-risk melanoma.

A PPI network constructed from the 158 senescence-associated downregulated genes comprised 107 nodes and 208 edges (Figure 3C). Network centrality analysis revealed several highly connected and influential genes, including *HMMR*, *BUB3*, *DEK*, *HNRNPH1*, *CBX3*, *XPO1*, *EWSR1*, *HNRNPA2B1*, *TRA2B*, and *SRSF3* (Figure 3D). Among the microRNA-predicted targets, Synaptotagmin Binding Cytoplasmic RNA Interacting Protein (SYNCRIP) displayed a hub-like profile characterized by high node degree, and Ring Finger Protein 138 (RNF138) occupied peripheral position within the network. These findings highlight SYNCRIP as the most topologically relevant microRNA-regulated candidate among the senescence-associated genes suppressed in high-risk melanoma.

### 3.5. Gene Ontology Enrichment Analysis

Gene Ontology enrichment analysis of the 158 senescence-associated genes downregulated in high-risk melanoma identified significant enrichment of biological processes related to RNA metabolism and gene expression. The most significantly enriched categories included RNA splicing (GO-ID 8380; adjusted *p* = 1.79 × 10^−5^), mRNA metabolic processing (GO-ID 6397; adjusted *p* = 2.13 × 10^−5^), mRNA metabolic process (GO-ID 16071; adjusted *p* = 7.27 × 10^−5^), RNA metabolic process (GO-ID 16070; adjusted *p* = 2.54 × 10^−5^), gene expression (GO-ID 10467; adjusted *p* = 8.36 × 10^−5^), RNA transport (GO-ID 50658; adjusted *p* = 3.62 × 10^−3^), RNA localization (GO-ID 6403; adjusted *p* = 3.99 × 10^−3^), and establishment of RNA localization (GO-ID 51236; adjusted *p* = 3.62 × 10^−3^) (Table 4).

Among the genes predicted to be regulated by melanoma-associated microRNAs, *SYNCRIP*, a predicted target of hsa-miR-425-5p, was present in multiple enriched categories related to RNA processing, RNA metabolism, and gene expression. *RNF138*, predicted to be targeted by hsa-miR-195-5p, was represented in categories related to cellular metabolic processes and regulation of biological processes. These results indicate that the two microRNA-predicted target genes are distributed across several of the enriched biological processes identified within the senescence-associated gene set.

## 4. Discussion

Cell cycle regulation is commonly used as an indicator of the biological effects of therapeutic interventions on tumor cells, including melanoma [62,63,64]. In general, cell cycle arrest is associated with reduced cellular proliferation and may consequently limit tumor progression [65,66,67]. Conversely, factors that promote cell cycle progression can contribute to uncontrolled proliferation, suggesting that their inhibition may represent a potential therapeutic strategy [68,69,70]. Several studies included in this review evaluated the effects of microRNAs on cell cycle progression and demonstrated that modulation of their expression frequently resulted in cell cycle arrest [30,31,51,52,54]. Notably, sustained cell cycle arrest is one of the hallmarks of CS, a phenomenon that has gained increasing attention in cancer biology because of its potential impact on tumor development and progression [71,72,73].

Cellular senescence is characterized by stable cell cycle arrest accompanied by distinct phenotypic and molecular alterations [74,75]. Commonly used markers for senescence characterization include increased SA-β-gal activity, upregulation of cell cycle inhibitors such as p16INK4a and p21CIP1, persistent DNA damage signaling, loss of Lamin B1, and chromatin remodeling [76,77,78]. Despite losing proliferative capacity, senescent cells remain metabolically active and can develop a characteristic secretory profile known as SASP, which influences neighboring and distant cells through the release of cytokines, growth factors, and other bioactive molecules [79,80,81]. Therefore, assessing senescence-related markers following microRNA-induced cell cycle arrest would provide important insights into the biological consequences of these interventions. However, only two studies [36,54] evaluated CS after demonstrating cell cycle arrest in melanoma cells, highlighting a major limitation of the current literature.

Previous studies have shown that senescence evolves over time and can result in distinct biological states [82,83,84]. During the early stages, often referred to as acute senescence, cells exhibit stable cell cycle arrest while displaying hallmark features of the senescent phenotype. Although the SASP can, in some contexts, exert detrimental effects, acute senescence is generally regarded as a beneficial process due to its roles in tissue repair, immune surveillance, and tumor suppression [85,86,87]. Consequently, the influence of these cells on the tumor microenvironment may be limited, potentially creating a therapeutic window during which senolytic agents could selectively eliminate senescent tumor cells before the accumulation of long-term senescence-associated effects [88,89]. This concept has led to the proposal of combining senescence induction with subsequent senolytic treatment as a strategy to suppress tumor growth while preventing the persistence of senescent cells [90,91,92].

In contrast, prolonged persistence of senescent cells can lead to the development of chronic senescence, characterized by a robust SASP profile [93,94]. Through the sustained secretion of cytokines, growth factors, proteases, and other bioactive molecules, these cells can remodel the tumor microenvironment, promoting inflammation, extracellular matrix remodeling, and the acquisition of more aggressive phenotypes by neighboring cells, thereby contributing to melanoma progression [1,95,96,97]. Therefore, the timing of senescence assessment represents a critical experimental variable, as analyses performed shortly after microRNA intervention may underestimate the establishment of the complete senescent phenotype.

Interestingly, the two studies that investigated CS evaluated the effects of microRNA modulation over relatively short experimental periods, both in vitro and in vivo [36,54]. Although these time frames are sufficient to characterize the early stages of senescence, they may not capture the long-term establishment of a chronic senescent phenotype. Consequently, the long-term fate of senescent melanoma cells remains poorly understood. This observation represents an important gap in melanoma research. CS is increasingly recognized as a relevant component of cancer biology because it can exert both tumor-suppressive and tumor-promoting effects. Future studies should incorporate extended follow-up periods and comprehensive senescence characterization to determine whether microRNA-induced cell cycle arrest results in transient growth suppression or the establishment of persistent senescent states capable of influencing melanoma progression.

A total of 5541 genes were found to be downregulated in high-risk melanoma, while 734 genes were reported as underexpressed during CS. The intersection of these datasets revealed 158 common genes. These findings suggest that components of senescence-associated transcriptional programs are suppressed in aggressive melanoma and may contribute to disease progression, indicating a potential impairment of cellular senescence mechanisms in advanced disease.

Target prediction analysis revealed that only two of the fifteen melanoma-associated microRNAs identified in the systematic review presented predicted targets within the 158-gene intersection. Specifically, hsa-miR-195-5p, and hsa-miR-425-5p were predicted to regulate *RNF138*, and *SYNCRIP*, respectively. The limited overlap observed between melanoma-associated microRNAs and senescence-associated genes suggests that this regulatory network remains largely unexplored.

The biological relevance of these findings is supported by evidence from other tumor models. Hsa-miR-195-5p has been reported to induce cell cycle arrest and senescence-like phenotypes in several cancer types, including breast, colorectal, and hepatocellular carcinomas, suggesting a conserved role in the control of cellular proliferation [98,99,100,101,102]. In contrast, hsa-miR-425-5p has been linked to more aggressive tumor phenotypes and poor clinical outcomes in different malignancies, although its role appears to be context dependent [103,104,105]. Collectively, these observations support the hypothesis that the two microRNAs identified in the present analysis may participate in molecular pathways connecting senescence regulation and tumor progression. Nevertheless, their specific contribution to senescence-associated mechanisms in melanoma remains largely unexplored and warrants further investigation.

In melanoma, among the identified targets, SYNCRIP emerged as the most relevant candidate. Network analysis demonstrated that SYNCRIP displayed a hub-like profile within the PPI network, indicating a potentially central role in senescence-associated molecular interactions. SYNCRIP is an RNA-binding protein that interacts with multiple protein complexes and participates in the post-transcriptional regulation of diverse cellular processes, including cell proliferation [106], epithelial–mesenchymal transition [107], and cell cycle regulation [108]. To date, no evidence has linked SYNCRIP to cellular senescence. In the present study, computational target prediction identified SYNCRIP as a potential target of hsa-miR-425-5p and as a gene downregulated in high-risk melanoma. Because hsa-miR-425-5p has been reported as a tumor suppressor in melanoma but as a pro-tumorigenic microRNA in several other malignancies, the biological significance of the predicted miR-425-5p–SYNCRIP interaction remains uncertain. Consequently, the observed downregulation of SYNCRIP in high-risk melanoma cannot currently be interpreted as supporting either biological model. Rather, these findings suggest that the proposed miR-425-5p–SYNCRIP regulatory axis represents a plausible candidate mechanism linking microRNA-mediated regulation to melanoma progression, but one that should be regarded as hypothesis-generating and requires functional validation in melanoma-specific experimental systems.

In contrast, predicted *RNF138* occupied more peripheral position but remain biologically relevant due to their involvement in processes such as transcriptional regulation, DNA damage responses, and tumor progression across different cancer types [109,110,111].

Taken together, the findings of this review suggest that microRNA-mediated regulation of cell cycle progression may represent an important mechanism influencing melanoma biology, although its relationship with CS remains insufficiently characterized. The predominance of studies reporting cell cycle arrest following microRNA modulation, combined with the limited assessment of senescence-associated markers, indicates that the long-term biological consequences of these interventions remain largely unknown. This gap is particularly relevant given the dual role of senescence in cancer, acting either as a tumor-suppressive barrier or as a contributor to tumor progression depending on its persistence and phenotypic evolution [57,61].

To explore potential links between microRNAs, senescence, and melanoma aggressiveness, an integrative in silico approach was employed, leading to the identification of a small subset of candidate genes potentially connecting these processes. Although hsa-miR-195-5p, hsa-miR-425-5p, and their predicted targets emerged as promising candidates, these findings should be interpreted with caution. The present analysis assumes that genes underexpressed in high-risk melanoma may be regulated, at least in part, by melanoma-associated microRNAs; however, changes in gene expression can arise from multiple regulatory mechanisms, including epigenetic alterations, transcriptional regulation, chromatin remodeling, genomic alterations, and post-transcriptional processes independent of microRNA activity. Therefore, the identified interactions should be considered hypothesis-generating rather than evidence of direct regulatory relationships. Nevertheless, the convergence of senescence-associated genes, high-risk melanoma transcriptomic profiles, microRNA target prediction, and network topology analysis provides a framework for prioritizing candidate molecules for future investigations. More broadly, our findings highlight that the relationship between microRNA-induced cell cycle arrest and CS remains an underexplored area in melanoma research. Experimental validation of the identified regulatory axes, together with longitudinal assessments of senescence phenotypes, may contribute to a better understanding of how microRNAs influence the balance between tumor suppression and tumor promotion during melanoma progression.

Collectively, these findings indicate that melanoma-associated microRNAs are consistently associated with the regulation of melanoma cell cycle progression; however, current evidence remains insufficient to conclude that most of these microRNAs directly induce cellular senescence. Future studies should combine cell cycle analyses with established senescence biomarkers to distinguish senescence from other non-proliferative states, including quiescence, terminal differentiation, and reversible growth arrest.

## 5. Conclusions

This systematic review demonstrates that while microRNAs frequently induce cell cycle arrest in melanoma, the relationship between microRNA activity and CS remains critically underexplored. Only two of fifteen included studies assessed senescence markers, representing a major knowledge gap. Integrative bioinformatics, used for hypothesis-generating, identified *SYNCRIP* as a hub gene linking microRNA regulation to senescence-associated transcriptional suppression in high-risk melanoma, warranting further experimental validation. Future investigations should prioritize longitudinal characterization of senescent phenotypes and evaluate the therapeutic potential of senescence-inducing microRNAs combined with senolytic agents.

## Figures and Tables

**Table 1:** Search terms, boolean operators, and eligibility filters applied in each database for the identification of studies included in the systematic review.

Database	Search Strategy	Filters Applied
PubMed	(melanoma OR “melanoma cells”) AND (microRNA OR miRNA) AND (“tumor progression” OR “tumor growth” OR “cancer progression”)	Document type: Adaptive Clinical Trial, Case Reports, Classical Article, Clinical Study, Clinical Trial, Clinical Trial, Phase I, Clinical Trial, Phase II, Clinical Trial, Phase III, Clinical Trial, Phase IV, Comparative Study, Controlled Clinical Trial, Equivalence Trial, Evaluation Study, Historical Article, Legal Case, Multicenter Study, Newspaper Article, Observational Study, Pragmatic Clinical Trial, Randomized Controlled Trial, Research Support, American Recovery and Reinvestment Act, Research Support, N.I.H., Extramural, Research Support, N.I.H., Intramural, Research Support, Non-U.S. Gov’t, Research Support, U.S. Gov’t, Research Support, U.S. Gov’t, Non-P.H.S., Research Support, U.S. Gov’t, P.H.S., Technical Report, Twin Study, Validation Study
Scopus	(TITLE-ABS-KEY (melanoma) OR TITLE-ABS-KEY (“melanoma cell *”) AND TITLE-ABS-KEY (microrna *) OR TITLE-ABS-KEY (mirna *) AND TITLE-ABS-KEY (“tumor progression”) OR TITLE-ABS-KEY (“tumor growth”) OR TITLE-ABS-KEY (“cancer progression”))	Document type: Article, Short survey; Language: English; Keyword: melanoma
Embase	(‘melanoma’ OR ‘melanoma cell’) AND (‘microRNA’ OR ‘miRNA’) AND (‘tumor growth’ OR ‘tumor progression’ OR ‘cancer progression’)	Publication typer: Article, Article in Press, Short Survey, Clinical trial; Disease: melanoma, uveal melanoma, cutaneos melanoma, metastatic melanoma
Dimensions	(melanoma OR “melanoma cells”) AND (microRNA OR miRNA) AND (“tumor progression” OR “tumor growth” OR “cancer progression”)	Publication type: article

Note: The asterisks (*) added to the selected search keywords indicate the use of wildcard truncation to retrieve variations of the root term (e.g., plural forms and other word variants).

**Table 2:** Summary of studies investigating microRNA function in melanoma.

Article	MicroRNA(s)	Experimental Approach	Biological Model	Main Assessments	Main Biological Effects
[51]	Hsa-miR-200a	Ex vivo + in vitro	Tissues (BN, PM, MM), cell lines (HEMn-DP, HEMaLP, AB-0301, AJL-0714, UCLA-SO-M14, GD-0645, UCLA-SO-M-219, SR-0078, LP-0024, EB-1075, MH-0331, TG-0873, SG0035, CS-0169, MB-1133, UCLA-SO-M-204, VN-0326, UCLA-SO-M-15, JT-1045, HM0525-ME, GA-0109, ML-0817, UCLA-SO-M-20, JH-1173, BG-0548, UCLA-SO-M-211, DP-0574-ME, BD-0548, UCLA-SO-M-16, WP-0614, MG-0471, IM-0223)	RT-qPCR, RNA-seq, miR transf., LRA, CDK6 OE clones, CC, viability, CF, 3D spheroids, ind. immuno., WB, CpG meth. prof., DNA demeth., palbociclib	Suppression of cell proliferation
[52]	Hsa-miR-664	In vitro + in vivo	Cell lines (A375.S2, A7, MeWo, RPMI-7951, SK-MEL-5, SK-MEL-24, SK-MEL-28), nude mice	MiR transf., WB, RT-qPCR, viability, CF, CC, TXA, LRA	Suppression of cell proliferation
[53]	Hsa-miR-425; hsa-miR-489	In vitro	Cell lines (A375, SK-MEL-2)	RIP, miR transf./depletion	Promotion of cell Proliferation
[54]	Hsa-miR-125b	Ex vivo + in vitro + in vivo	Tissues (PM, MM), cell line (Mel-Juso), BALB/c nude mice	ISH, miR transf., RT-qPCR, RNA-seq, CF, CC, SA-β-gal, WB, apoptosis, TXA	Suppression of cell proliferation
[31]	Hsa-miR-590-5p	In vitro + in vivo	Cell lines (A2058, A375, HEMa-LP, 293), BALB/c nude mice	MiR transf./depletion, RT-qPCR, viability, CC, apoptosis, WB, TXA, LRA	Suppression of cell proliferation
[30]	Hsa-miR-344d-3-5p	In vitro + in vivo	Cell line (B16), C57BL/6 mice	RNA-seq, miR transf./depletion, LRA, RNA PD, viability, CC, WB, RT-qPCR, STM	Suppression of cell proliferation
[55]	Hsa-miR-507	Ex vivo + in vitro	Tissues (BN, PM, MM), cell lines (A375, SK-MEL-2)	MiR transf., RT-qPCR, viability, invasion, CC, LRA, RIP, WB	Suppression of cell proliferation
[36]	Hsa-miR-194-5p	In vitro	Cell lines (Mel Wei, Mel Im)	MiR transf., LRA, WB, RT-qPCR	Suppression of cell proliferation
[56]	Hsa-miR-195	Ex vivo + in vitro	Tissues (PM, MM), cell lines (SK-Mel-28, SK-Mel-147	RT-qPCR, miR transf./depletion, WB, LRA, CC, viability, migration/invasion	Promotion of cell proliferation
[57]	Hsa-miR-30a-5p	In vitro	Cell lines (M8, SK-Mel-19, HEK293T)	MiR transf./depletion, viability, RT-qPCR, WB, LRA, microarrays, cisplatin	Promotion of cisplatin resistance
[58]	Hsa-miR-876-3p	Ex vivo + in vitro + in vivo	Tissues (BN, PM), cell lines (C8161.9, A375, LOX, 1205-Lu, Ma-Mel-12, HEM), nude mice	RT-qPCR, RNA-seq, miR transf., LRA, viability, CF, CC, migration/invasion, apoptosis, WB, vemurafenibe, TXA	Suppression of cell proliferation
[59]	Hsa-miR-15a	In vitro + in vivo	Cell lines (A375, SK-MEL-28, WM2552C, B16-F10), C57BL/6 mice	MiR transf., viability, CC, migration/invasion, WB, TXA, LRA	Suppression of cell proliferation
[60]	Hsa-miR-425	Ex vivo + in vitro	Tissues (N, M), cell lines (SK-MEL-28, UACC257, A375, WM-115, HEM)	RT-qPCR, miR transf., viability, migration/invasion, WB, CF, CC, LRA	Suppression of cell proliferation
[61]	Hsa-miR-625-5p	Ex vivo + in vitro + in vivo	Tissues (N, M), cell lines (A-375, HT-144, SK-MEL-1, A2058, HEM), BALB/c nude mice	RT-qPCR, RIP, miR transf., LRA, viability, CF, apoptosis, CC, migration/invasion, WB, TXA, cisplatin	Suppression of cell proliferation
[50]	N/A	In vitro	EVs released by eosinophils activated by IL-33 (Eo5, Eo33)	RT-qPCR, RNA-seq	EVs suppression of cell proliferation

Note: Only methods and biological effects directly related to microRNA investigation are reported. Abbreviations: BN, benign nevi; CC, cell cycle; CDK6 OE clones, stable CDK6-overexpressing clones; CF, colony formation; CpG meth. prof., epigenome-wide CpG methylation profiling; DNA demeth., DNA demethylation assay; HEM, human epidermal melanocytes; ind. immuno, indirect immunofluorescence; ISH, in situ hybridization; LRA, luciferase reporter assay; M, melanoma; miR, microRNA; miR transf., microRNA transfection; MM, metastatic melanoma; N, normal; PM, primary melanoma; RIP, RNA immunoprecipitation; RNA PD, RNA pull-down; RNA-seq, RNA sequencing; RT-qPCR, reverse transcription quantitative polymerase chain reaction; SA-β-gal, senescence-associated β-galactosidase; STM, syngeneic tumour model; TXA, tumour xenograft assay; WB, Western blot.

**Table 3:** Risk-of-bias evaluation of the included studies.

Study	Score	Risk of Bias	Reasons for Score Reduction
[51]	0.71	Low	No cell line authentication or mycoplasma testing
[52]	0.36	Moderate	Limited methodological reporting, no cell line authentication or mycoplasma testing, and limited in vivo reporting
[53]	0.21	High	Inappropriate controls, limited in vitro reporting, and no in vivo methodological details
[54]	0.47	Moderate	Inappropriate controls, no cell line authentication or mycoplasma testing, and incomplete in vivo reporting
[31]	0.57	Moderate	Lack of cell line authentication and incomplete reporting of in vivo methods
[30]	0.71	Low	Lack of cell line authentication, blinding, and reporting of animal losses in in vivo experiments
[55]	0.71	Low	Lack of cell line authentication and mycoplasma testing
[36]	0.62	Low	Insufficient methodological details, lack of cell line authentication, and absence of mycoplasma testing
[56]	0.43	Moderate	Inappropriate controls, insufficient methodological details, lack of cell line authentication, and absence of mycoplasma testing
[57]	0.71	Low	Lack of cell line authentication and mycoplasma testing
[58]	0.71	Low	Lack of cell line authentication, mycoplasma testing, baseline similarity reporting, and treatment blinding
[59]	0.5	Moderate	Inadequate experimental controls, lack of cell line authentication and mycoplasma testing, and limited in vivo reporting
[60]	0.71	Low	Lack of cell line authentication and mycoplasma testing
[61]	0.8	Low	Insufficient methodological information for reproducibility and absence of blinding in in vivo experiments
[50]	0.5	Moderate	Lack of cell line authentication and incomplete reporting of in vivo methods

**Table 4:** Significantly enriched GO biological processes identified by BiNGO analysis.

GO-ID	*p*-Value	Corrected *p*-Value	Description	Genes Set
6807	1.7878 × 10^−8^	1.7868 × 10^−5^	Nitrogen compound metabolic	*PRPS2*|*RPL5*|*TCERG1*|*ODC1*|*HMGB2*|*SLC1A3*|*HNRNPR*|*DBI*|*SMC3*|*ETS1*|***SYNCRIP*|***MRPL1*|*MED30*|*RSRC1*|*RAD21*|*TRA2B*|*DPYSL3*|*SNRPB2*|*TOPBP1*|*GUSB*|*GLUL*|*NOP58*|*NMI*|*LSM6*|*MTHFD2*|*HNRNPH1*|*FUBP1*|*HNRNPF*|*HNRNPA2B1*|*SRSF3*|*CEBPZ*|*HPRT1*|*HNRNPH3*|*SRSF7*|*PTMA*|*RBMX*
8380	3.0886 × 10^−8^	1.7868 × 10^−5^	RNA splicing	*HNRNPR*|***SYNCRIP*|***LSM6*|*RSRC1*|*HNRNPH1*|*HNRNPF*|*TRA2B*|*HNRNPA2B1*|*SRSF3*|*SNRPB2*|*HNRNPH3*|*SRSF7*|*RBMX*
34641	5.8073 × 10^−8^	2.1263 × 10^−5^	Cellular nitrogen compound metabolic process	*PRPS2*|*RPL5*|*TCERG1*|*ODC1*|*HMGB2*|*SLC1A3*|*HNRNPR*|*DBI*|*SMC3*|*ETS1*|***SYNCRIP*|***MRPL1*|*MED30*|*RSRC1*|*RAD21*|*TRA2B*|*DPYSL3*|*SNRPB2*|*TOPBP1*|*GLUL*|*NOP58*|*NMI*|*LSM6*|*HNRNPH1*|*FUBP1*|*HNRNPF*|*HNRNPA2B1*|*SRSF3*|*CEBPZ*|*HPRT1*|*HNRNPH3*|*SRSF7*|*PTMA*|*RBMX*
6397	7.3510 × 10^−8^	2.1263 × 10^−5^	mRNA metabolic processing	*HNRNPR*|***SYNCRIP*|***LSM6*|*RSRC1*|*HNRNPH1*|*HNRNPF*|*TRA2B*|*HNRNPA2B1*|*SRSF3*|*SNRPB2*|*HNRNPH3*|*SRSF7*|*RBMX*
16070	1.0970 × 10^−7^	2.5384 × 10^−5^	RNA metabolic process	*RPL5*|*TCERG1*|*NOP58*|*HNRNPR*|*NMI*|*ETS1*|***SYNCRIP*|***LSM6*|*MRPL1*|*MED30*|*RSRC1*|*HNRNPH1*|*FUBP1*|*HNRNPF*|*TRA2B*|*HNRNPA2B1*|*SRSF3*|*SNRPB2*|*CEBPZ*|*HNRNPH3*|*SRSF7*|*RBMX*
90304	2.0460 × 10^−7^	3.9453 × 10^−5^	Nucleotide and nucleic acid metabolic process	*RPL5*|*TCERG1*|*HMGB2*|*HNRNPR*|*SMC3*|*ETS1*|***SYNCRIP*|***MRPL1*|*MED30*|*RSRC1*|*RAD21*|*TRA2B*|*SNRPB2*|*TOPBP1*|*NOP58*|*NMI*|*LSM6*|*HNRNPH1*|*FUBP1*|*HNRNPF*|*HNRNPA2B1*|*SRSF3*|*CEBPZ*|*HNRNPH3*|*SRSF7*|*PTMA*|*RBMX*
6139	2.8191 × 10^−7^	4.6596 × 10^−5^	Nucleobase nucleoside	*PRPS2*|*RPL5*|*TCERG1*|*HMGB2*|*HNRNPR*|*SMC3*|*ETS1*|***SYNCRIP*|***MRPL1*|*MED30*|*RSRC1*|*RAD21*|*TRA2B*|*DPYSL3*|*SNRPB2*|*TOPBP1*|*NOP58*|*NMI*|*LSM6*|*HNRNPH1*|*FUBP1*|*HNRNPF*|*HNRNPA2B1*|*SRSF3*|*CEBPZ*|*HPRT1*|*HNRNPH3*|*SRSF7*|*PTMA*|*RBMX*
6396	5.0935 × 10^−7^	7.2716 × 10^−5^	RNA processing	*RPL5*|*NOP58*|*HNRNPR*|***SYNCRIP*|***LSM6*|*MRPL1*|*RSRC1*|*HNRNPH1*|*HNRNPF*|*TRA2B*|*HNRNPA2B1*|*SRSF3*|*SNRPB2*|*HNRNPH3*|*SRSF7*|*RBMX*
16071	5.6564 × 10^−7^	7.2716 × 10^−5^	mRNA metabolic process	*HNRNPR*|***SYNCRIP*|***LSM6*|*RSRC1*|*HNRNPH1*|*HNRNPF*|*TRA2B*|*HNRNPA2B1*|*SRSF3*|*SNRPB2*|*HNRNPH3*|*SRSF7*|*RBMX*
10467	7.2275 × 10^−7^	8.3623 × 10^−5^	Gene expression	*RPL5*|*TCERG1*|*NOP58*|*HNRNPR*|*NMI*|*ETS1*|***SYNCRIP*|***LSM6*|*MRPL1*|*MED30*|*RSRC1*|*HNRNPH1*|*FUBP1*|*HNRNPF*|*TRA2B*|*HNRNPA2B1*|*CASP1*|*SRSF3*|*SNRPB2*|*CEBPZ*|*HNRNPH3*|*SRSF7*|*PTMA*|*RBMX*
7059	1.4307 × 10^−5^	1.5049 × 10^−3^	Chromosome segregation	*PSRC1*|*RAD21*|*BUB3*|*SMC3*|*SMC4*|*SKA2*
51236	4.3836 × 10^−5^	3.6228 × 10^−3^	Establishment of RNA localization	*ENY2*|*XPO1*|*HNRNPA2B1*|*NUP54*|*NMI*|*NXT2*
50657	4.3836 × 10^−5^	3.6228 × 10^−3^	Nucleic acid transport	*ENY2*|*XPO1*|*HNRNPA2B1*|*NUP54*|*NMI*|*NXT2*
50658	4.3836 × 10^−5^	3.6228 × 10^−3^	RNA transport	*ENY2*|*XPO1*|*HNRNPA2B1*|*NUP54*|*NMI*|*NXT2*
6403	5.1691 × 10^−5^	3.9871 × 10^−3^	RNA localization	*ENY2*|*XPO1*|*HNRNPA2B1*|*NUP54*|*NMI*|*NXT2*
15931	9.5100 × 10^−5^	6.8769 × 10^−3^	Nucleobase nucleoside	*ENY2*|*XPO1*|*HNRNPA2B1*|*NUP54*|*NMI*|*NXT2*
9987	1.0253 × 10^−4^	6.9784 × 10^−3^	Cellular process	*RPL5*|*TCERG1*|*HMGB2*|*HNRNPR*|*DBI*|*SMC3*|*ETS1*|*SMC4*|*CHD1*|*AKAP12*|*PPP1CC*|***SYNCRIP*|***SMCHD1*|*MRPL1*|*XPO1*|*MIS18BP1*|*RSRC1*|*DPYSL3*|*CASP1*|*EMILIN2*|*EMILIN1*|*TOPBP1*|*HMGN2*|*GLUL*|*HOXA5*|*GAS2L3*|*POSTN*|*SERPINF1*|*TNFRSF1B*|*UCHL5*|*SFRP1*|*PSRC1*|*MTHFD2*|*HNRNPH1*|*HAT1*|*CDH11*|*PSME1*|*SRSF3*|*SUCLG2*|*NUP54*|*HPRT1*|*HNRNPH3*|*SRSF7*|*PTMA*|*FKBP5*|*PRPS2*|*ENY2*|*COL15A1*|*INSIG1*|*ODC1*|*SLC1A3*|*SKA2*|*PTGS1*|*GNG2*|*MED30*|*PRKAR2B*|*RAD21*|*TRA2B*|*USP1*|*BRIX1*|*SNRPB2*|*BUB3*|*SNX5*|*GSDMD*|*EGR1*|*NOP58*|*CBX3*|*NMI*|*CXCL12*|*LSM6*|*FUBP1*|*HNRNPF*|*APOC1*|*HNRNPA2B1*|*CEBPZ*|*RBMX*

Note: “*SYNCRIP*” is shown in bold in the Gene Set column, as it was identified as a hub gene.

## Data Availability

No new data were created or analyzed in this study. Data sharing is not applicable to this article.

## Supplementary Materials

The following supporting information can be downloaded at https://www.mdpi.com/article/10.3390/ijms27146462/s1.

## Abbreviations

The following abbreviations are used in this manuscript:

PRISMA	Preferred Reporting Items for Systematic Reviews and Meta-Analyses
CS	Cellular Senescence
SASP	Senescence-Associated Secretory Phenotype
EV(s)	Extracellular Vesicle(s)
lncRNA(s)	long non-coding RNA(s)
PICO	Population, Intervention, Comparison, Outcome
N.I.H.	National Institutes of Health
U.S. Gov’t	United States Government
P.H.S.	Public Health Service
ABS	Abstract
KEY	Keywords
DEG(s)	Differentially Expressed Gene(s)
SKCM	Skin Cutaneous Melanoma
CSRS	Cellular Senescence-Related Signature
TCGA-SKCM	The Cancer Genome Atlas Skin Cutaneous Melanoma Cohort
LASSO Cox	Least Absolute Shrinkage and Selection Operator Cox Proportional Hazards Model
FDR	False Discovery Rate
log_2_FC	log_2_ Fold Change
PPI	Protein–Protein Interaction
BiNGO	Biological Networks Gene Ontology Tool
GO	Gene Ontology
HB	Hub-Bottleneck
Cont.	Continuation
BN	Benign Nevi
CC	Cell Cycle
CDK6 OE clones	Stable CDK6-overexpressing Clones
CF	Colony Formation
CpG meth. prof.	Epigenome-wide CpG Methylation Profiling
DNA demeth.	DNA Demethylation Assay
HEM	Human Epidermal Melanocytes
ind. immuno	Indirect Immunofluorescence
ISH	In Situ Hybridization
LRA	Luciferase Reporter Assay
M	Melanoma
miR	MicroRNA
miR transf.	MicroRNA Transfection
MM	Metastatic Melanoma
N	Normal
PM	Primary Melanoma
RIP	RNA Immunoprecipitation
RNA PD	RNA Pull-down
RNA-seq	RNA Sequencing
RT-qPCR	Reverse Transcription Quantitative Polymerase Chain Reaction
SA-β-gal	Senescence-associated β-galactosidase
STM	Syngeneic Tumour Model
TXA	Tumour Xenograft Assay
WB	Western Blot
SYNCRIP	Synaptotagmin Binding Cytoplasmic RNA Interacting Protein
ETS1	ETS Proto-Oncogene 1, Transcription Factor
RNF138	Ring Finger Protein 138
CAPES	Coordination for the Improvement of Higher Education Personnel (Brazil)
DS	Demanda Social
UFPR	Federal University of Paraná (Brazil)
UFPR/R/PRPI/COFPI	Federal University of Paraná/Rector’s Office/Pro-Rectorate for Research and Innovation/Coordination of Research and Innovation Funding
