# Supplementary material for: MicroRNAs and Cellular Senescence in Melanoma: An Underexplored Link to Tumor Progression—A Systematic Review with Bioinformatics Analyses
Source: Int J Mol Sci. 2026 Jul 21;27(14):6462. doi: 10.3390/ijms27146462 (PMC13410509; doi:10.3390/ijms27146462)
Supplement: Supplementary file 1 [file ijms-27-06462-s001.zip › Table S1 - Supplementary Material.xlsx - Planilha1.pdf]

DOI	Authors	Year	Article title	Journal	Country and institution	Experimental model	Sample size	Cell lines/samples	Intervention/Exposure	Assessment methods	Biological markers	Main findings	Conclusions	Risk of bias/Conflicts of interest	Additional observations
[51] 10.1016/j.jid. 2017.03.039	Matias A. Bustos, Shigeshi Ono, Diego M. Marzese, Takashi Oyama, Yuuki Iida, Garrett Cheung, Nellie Nelson, Sandy C. Hsu, Qiang Yu, Dave S.B. Hoon	2017	MiR-200a Regulates CDK4/6 Inhibitor Effect by Targeting CDK6 in Metastatic Melanoma	Society for Investigative Dermatology Impact Factor 5.7	Department of Translational Molecular Medicine, Division of Molecular Oncology, John Wayne Cancer Institute at Providence Saint John's Health Center, Santa Monica, California, USA	Ex vivo	n = 75	Archival paraffin-embedded human tissues. Nevi = 10, primary melanoma = 7, lymph nodes = 33, distant organs = 25	N/A	qRT-PCR	Expression of miR-200a	Decreased levels of miR-200a in melanoma compared to the primary tumor	- miR-200a induces a decrease in CDK6, which consequently reduces p-Rb1 levels. In this way, p-Rb1 represses the transcriptional activity of E2F1 and blocks signaling in these pathways, leading to a reduction in the targets of this transcription factor;<br>- Overexpression of miR-200a reduces melanoma cell proliferation and tumor-forming ability;<br>- When CDK6 is already elevated, miR-200a is unable to regulate it;<br>- miR-200a has an effect on the growth of metastatic melanoma that can be rescued by CDK6 overexpression;<br>- Metastatic melanoma cells are sensitive to palbociclib treatment.	The authors declared that they had no conflicts of interest.	- miR-200a inhibits CDK6, reducing proliferation. But doesn't that lead to senescent cells that could worsen the patient's prognosis?<br>- No tests were performed to confirm senescence, so could the cells be in quiescence?<br>- Could this increase in cells arrested in G0 and G1 by palbociclib be senescence, which would lead to a worse long-term prognosis for the patient?
							n = 32								
						In vitro	N/A	Cell culture (BD-0548, DP-0574 and WP-0614)	5-aza-2'-deoxycytidine DNA methyltransferase inhibitor			The expression of miR-200a increased by 3 to 8 times			
									Transfection with miR-200a or miR-ctrl	RNA sequencing	Gene expression	Identification of 390 different genes expressed following miR-200a transfection, of which 143 were downregulated			
						In silico		Target genes of miR-200a	N/A	Prediction algorithms: DIANA-microT, RNA22, and TargetScan	Targets of miR-200a	63 of the 143 down-regulated genes are targets of miR-200a, with 5 of them being consistently identified across all databases (ACOT7, ANP32E, CDK6, EPN1, and LRRTM2)			
						In vitro		Cell culture (DP-0574, HM-0525, and WP-0614)	Transfection with miR-200a or miR-ctrl	qRT-PCR, Western blotting, and indirect immunofluorescence	CDK6 Expression / p-Rb1 and Rb1 Activity	miR-200a reduced CDK6 expression, thereby reducing cell proliferation / Rb1 and p-Rb1 are found in the nucleus and decrease in the absence of CDK6 and in the presence of increased miR-200a			
										Flow cytometry	Cell cycle phase	Increase in cells arrested in the G0 and G1 phases of the cell cycle			
										Microscopy	Cell count	Decreased cell proliferation when miR-200a is upregulated; reduced ability to form spheroids after 9 days			
								Melanocyte cells, LNM, and DOM	CDK6 expression	WB, qRT-PCR	CDK6	Elevated CDK6 levels in metastatic melanoma cells compared to primary melanoma cells			
						In silico	n = 125	Patient data from the TCGA	Correlation between CDK6 and miR-200a	Correlation charts	N/A	There was no correlation			
						In vitro	N/A	Cell culture (M-204 and LP-0024)	CDK6, CDK4, p-Rb1 and Rb1	WB	CDK6 expression levels	When CDK6 levels are very high, increased expression of miR-200a does not significantly alter its expression			
								Two clones with overexpression of CDK6 (WP-C2 and WP-C3) and one clone with low expression of CDK6 (WP-C1)	miR-200a		p-Rb1 expression levels	p-Rb1 remained unchanged in WP-C3 but decreased in WP-C1. Endogenous CDK6 levels were reduced in both WP-C1 and WP-C3.			
								Melanoma cells	1–5,000 nmol/L of palbociclib for 72 hours	CellTiter-Glo®	Cell viability	100 nmol/L is half the maximum inhibitory concentration for palbociclib in metastatic melanoma cells			
									Palbociclib	WB	Phosphorylation status of p-Rb1 and spheroid formation	The treatment reduced Rb1 and p-Rb1 levels and the size of the spheroid, thereby inhibiting cell growth			
								Melanoma cells transfected with miR-200a and miR-ctrl		WB and flow cytometry	CDK4/6 expression	CDK6 levels decreased, but CDK4 levels did not. Palbociclib increased the percentage of cells arrested in the G0 and G1 phases			
						In vitro	N/A	Cell cultures of A375.S2, A7, MeWo, RPMI-7951, SK-MEL-5, SK-MEL-24, and SK-MEL-28	N/A	RT-PCR	miR-644 expression	miR-664 is downregulated in melanoma tissues	- Upregulation of miR-664 may inhibit melanoma cell tumorigenesis in vitro;		
									Transfection with the hsa-miR-664mimic oligonucleotide	Microscopy and MTT		Overexpression of miR-664 drastically reduced cell growth and cell adhesion			

[52] 10.1097/MD.0000000000001327	Zhenhua Ding, Sun Jian, Xuebiao Peng, Yimin Liu, Jianyu Wang, Li Zheng, Chengshan Ou, Yinghui Wang, Weixia Zeng, Meijuan Zhou	2015	Loss of MiR-664 Expression Enhances Cutaneous Malignant Melanoma Proliferation by Upregulating PLP2	Medicine (Baltimore) Impact Factor 4.2	Department of Radiation Medicine (ZD, LZ, CO, YW, MZ), School of Public Health and Tropic Medicine, Southern Medical University,Guangzhou, Guangdong,China			Cell culture of A375.S2 and SK-MEL-28 CMM	BrdU incorporation	Immunofluorescence and flow cytometry		The number of cells in the S phase decreased, and these cells overexpressed miR-664. There was an increase in the percentage of cells in the G1/G0 phase	[illegible] - miR-664 induces G1/S arrest in melanoma cells; - The reduction in tumor growth is due to miR-664, as it regulates cell entry into the G1/S phase; - miR-664 acts on cell proliferation by targeting PCNA; - miR-664 specifically affects the 3'UTR region of PLP2; - miR-664 inhibits melanoma cell proliferation by suppressing PLP2; - Overexpression of miR-664 led to an increase in the CDK inhibitor p21 in melanoma cells; - Overexpression of miR-664 resulted in a decrease in the cell cycle regulator cyclin D1 in melanoma cells.	The authors have no conflicts of interest to disclose.	There was no test for senescence. How can we tell if the cells that have stopped in the cell cycle are senescent or quiescent?
									miR-644 inhibitor	Microscopy and flow cytometry	Cell growth and cell cycle phase	There was an increase in cell proliferation and in the number of colonies			
						In vivo	n = 10 (2	Nude mice	Subcutaneous injection	Serial measurements	Tumor volume by	The smaller tumors were			
						In vitro	N/A	Cell culture of	Transfection of miR-	WB	PCNA expression	PCNA expression decreased			
						In silico		miR-664	N/A	TargetScan, Pictar,	PCNA expression	They are inversely			
						In vitro		Cell culture of A375.S2 and SK-MEL-28 CMM	miR-664 Transfection	WB	GFP-y-tubulin	The cells inhibited GFP			
									Transfection of miR-	Subcloning	Inhibitory activity of	The suppressive effect of			
									Transfection of miR-664 and PLP2 with and without the 3'UTR region	WB and flow cytometry	PLP2 protein expression and cell growth rate	Cells expressing PLP2 without a 3'UTR showed increased PLP2 expression and a higher growth rate. The percentage of cells in the S phase also increased, while the percentage of cells in the G0/G1 phase decreased.			
[53] PMCID: PMC5250706	Xiangjun Chen, Hao Dong, Sha Liu, Li Yu, Dexiong Yan, Xingwei Yao, Weijing Sun, Dezhi Han, Guozhen Gao	2017	Long noncoding RNA MHENCR promotes melanoma progression via regulating miR-425/489-mediated PI3K-Akt pathway	American Journal of Translational Research Impact Factor 4.06	Department of Burn and Plastic Surgery, The 253rd Hospital of PLA, Hohhot, Inner Mongolia, China	Ex vivo	n = 50 (30 malignant melanoma tissues and 20 matched normal skin controls)		N/A	qPCR	Expression of MHENCR	Higher expression of MHENCR in melanoma tissues; As the stages of melanoma progressed, MHENCR expression increased	- MHENCR expression is elevated in melanoma tissues and even higher in advanced stages and metastatic lesions; - High MHENCR expression is associated with a poor prognosis in patients with melanoma; - Downregulation of MHENCR reduces melanoma cell proliferation and induces cell cycle arrest and apoptosis; - Downregulation of MHENCR reduced the motility of melanoma cells; - MHENCR is physically associated with miR-425 and miR-489 but was not degraded by them, suggesting that MHENCR functions as a competing endogenous RNA for these microRNAs; - MHENCR activates the IGF1- and SPIN1-mediated pathway through association with miR-425 and miR-489; - Downregulation of MHENCR inhibits the growth and metastasis of melanoma cells.	The authors have no conflicts of interest to disclose.	- There is a significant lack of information regarding the methods used in each experiment. The authors merely discuss the results; - Decreased MHENCR induces cell cycle arrest, but no tests were conducted to confirm whether this is reversible or not; - The experiments are quite confusing when compared to those in previous articles.
							n = 16 pairs (primary melanoma tissues and matched metastatic lymph node lesions)					Higher expression of MHENCR in metastatic tissues			
												The higher the expression,			
						In vitro	N/A	Cell culture (A375 and SK-MEL-2)		RACE	MHENCR sequencing	N/A			
										Subcellular	Location of	Located predominantly in the			
									2 shRNAs	RT-qPCR	Reduction of	shRNA 2 and 3 were able to			
										CCK-8	Cell proliferation	A reduction in MHENCR			
										Flow cytometry	Cell cycle	A reduction in MHENCR			
										TUNEL	Cell apoptosis	Apoptosis was induced due			
										Transwell assays	Cell migration	Decreased cell migration			
						In silico	N/A	N/A		miRcode	Prediction of	Two binding sites on miR-			
						In vitro		Cell culture (A375 and SK-MEL-2 cells overexpressing miR-425 or miR-489)	2 mutated MHENCR control sequences	RIP tests using the MS2 vector system	Potential competitors	Specific binding of MHENCR to miR-425 and miR-489			
									Anti-AGO	RIP		The overexpression of miR-			
									N/A	RT-qPCR	Expression of	MiR-425 and miR-489 did			
									2 shRNAs	qPCR and WB	Effect of MHENCR	The reduction in MHENCR,			
										qPCR	Decrease in miR-	The suppression of IGF1			
											Decrease in miR-	The suppression of SPIN1			
										WB	Effect of MHENCR	Reduction of MHENCR			
						In vivo	Not specified	BALB/c nude mice	Subcutaneous injection of stable, controlled MHENCR cells derived from A375 into nude mice	Weight and size	Tumor size with and without shRNAs	When MHENCR was downregulated by shRNA, subcutaneous growth of the xenografts was inhibited			
										qPCR	Expression of IGF1	Downregulation of IGF1 and			
									Injection of A375 cells	Luciferase signal	Progression of	Downregulation of MHENCR			
[54] 10.1186/1471-5945-14-8	Anne Marie Nyholm, Catharina M Lerche, Valentina Manfé, Edyta Biskup, Peter Johansen, Niels Morling, Birthe Mørk Thomsen, Martin Glud, Robert	2014	miR-125b induces cellular senescence in malignant melanoma	BMC Dermatology Impact Factor 3.08 Archived journal	Department of Dermatology, Faculty of Health and Medical Sciences, University of Copenhagen, Bispebjerg Hospital, Copenhagen, Denmark	Ex vivo	n = 17 (15 superficial spreading melanomas and 2 nodular melanomas)	N/A		ISH	Analysis of miR-125b	Expressed in both primary tumors and lymph node metastases; Predominantly localized in the nuclei; Larger cells appeared to express higher levels of miR-125b;	- miR-125b acts as a tumor suppressor in cutaneous melanoma by regulating cellular senescence; - miR-125b induces senescence focally in tumors, and its effect on tumor mass is negligible.	The authors declare that they have no competing interests.	- The article is about senescence, but it views senescence as beneficial for halting tumor progression without analyzing chronic senescence; - “miR-125b found predominantly in the nuclei.” Does it act on mRNAs even before they enter the cytoplasm? They do not explain this finding; - They did not include comparison images regarding apoptosis; - In the in vivo model, miR-125b was observed in the cytoplasm. There was no explanation for this
						In vitro	N/A	Cell culture (Mel-Juso)	Transfection with miRVec-125b	RT-qPCR	Expression of miR-	Sequencing confirmed that			
										Microscopy	Cell observation	They formed fewer and			
										Flow cytometry	Stages of the cell	Cell cycle arrest at the			
										Microscopy	Cell observation	Enlarged, flattened cells with			
										SA-beta-gal staining	Evidence of	Increased expression of SA-			
										WB	Markers of	Upregulation of the p53, p21,			
										Comparison of Annexin A-positive cells with transfected cells	Cell apoptosis	No evidence of apoptosis			

	Glad, Robert Gniadecki					In vivo	n = 6 male	BALB/C nude mice	Mel-Juso cells infected subcutaneously	Macroscopic and histological examination of lung and liver tissue	Tumor formation	Within 4 weeks, subcutaneous tumors formed, but there were no distant metastases			There was no explanation for this finding;<br>- They did not test other cell models because the vector generated a very strong apoptotic response. So to what extent could this study be replicated?
										ISH	miR-125b expression	Significant expression of			
										RT-qPCR		Stability of miR-125b			
										ISH and X-gal staining	Senescence	Reduced mitotic activity and			
										Visual assessment of	Quantification of	Decreased expression of the			
										Weighing	Tumor weight	There was no difference in			
[31] 10.3892/or. 2018.6633	Kuanhou Mou, Meiling Ding, Dan Han, Yan Zhou, Xin Mu, Wenli Liu, Lijuan Wang	2018	miR-590-5p inhibits tumor growth in malignant melanoma by suppressing YAP1 expression	Oncology Reports Impact Factor: 3.9	Department of Dermatology, The First Affiliated Hospital of Xi'an Jiaotong University, Xi'an, Shaanxi	In vitro	N/A	Cell culture: Normal human cells and A375 cells	N/A	RT-qPCR	miRNA screening	Reduced levels of miR-590-5p in A375 cells; Table listing the miRNAs detected	- miR-590-5p is downregulated in metastatic melanoma cells and can suppress cell proliferation and tumor growth;<br>- YAP1 is upregulated and inversely associated with the expression of miR-590-5p;<br>- YAP1 is a direct target and functional mediator of miR-590-5p;<br>- miR-590-5p may function as a tumor suppressor gene in metastatic melanoma;<br>- miR-590-5p exerted antitumor roles;<br>- YAP1 was identified as an oncogene and was highly expressed in metastatic melanoma cells;<br>- Silencing YAP1 can restore the inhibitory effects of miR-590-5p on the cell cycle and tumor growth.	The authors declare that they have no competing interests.	The charts are poorly designed. It is very difficult to see and distinguish between the dots and squares in the line charts and the patterns in the bar charts.
								Cell culture (A2058 and A375)	Transfection of miR-590-5p mimics into A2058 cells and miR-590-5p inhibitors into A375 cells	CCK8	Cell proliferation	In A2058, proliferation was			
										Flow cytometry	Cell apoptosis	Increased apoptosis in			
											Cell cycle	A2058 showed an increase			
						In vivo	n=5 for each	Balb/C female	Injection of	Tumor measurement	Tumor size	miR-590-5p inhibits tumor			
						In silico	N/A			Unbiased computational screening (TargetScan, PicTar, and miRNAda)	Identify potential targets of miR-590-5p	YAP1 contains a potential target site for miR-590-5p and was selected			
						In vitro	N/A	Normal cells	Co-transfection with YAP1 WT 3'-UTR mimics and miR-590-5p	Luciferase reporter assays	Luciferase activity	293 cells showed a >60% decrease in luciferase activity, while the other half showed no significant effects			
								Cell culture (Normal cells, A2058, and A375)		RT-qPCR and Western blot	YAP1 expression	YAP1 expression was			
											YAP1 mRNA expression	YAP1 mRNA levels were reduced and YAP1 protein expression was decreased in A2058 cells transfected with miR-590-5p mimics; YAP1 mRNA levels and YAP1 protein expression were increased in A375 cells transfected with miR-590-5p inhibitors			
								Cell culture (A375)	Transfection of YAP1 siRNA or NC and miR-590-5p inhibitors	Flow cytometry	Cell apoptosis and cell proliferation	Attenuation of the effects of cell proliferation; Cells treated with siRNA inhibitors and miR-590-5p exhibited increased apoptosis; Silencing YAP1 protects against the effects of miR-590-5p inhibitors on cell cycle progression and tumor growth			
[30] 10.1038 /s41419-022-04736-6	Dan Wang, Jianfei Chen, Bohan Li, Qingling Jiang, Ling Liu, Ziyi Xia, Qiusheng Zheng, Minjing Li, Defang Li	2022	A noncoding regulatory RNA Gm31932 induces cell cycle arrest and differentiation in melanoma via the miR-344d-3-5p/Prc1 (and Nuf2) axis	Cell Death & Disease Impact Factor 9.6	Yantai Key Laboratory of Pharmacology of Traditional Chinese Medicine in Tumor Metabolism, School of Integrated Traditional Chinese and Western Medicine, Binzhou Medical University, Yantai	In vitro	N/A	Cell culture (B16 - mouse melanoma)	Cell induction by ATRA and PB-4	CCK8 and microscopy	Cell proliferation rate	Decreased cell proliferation in B16 cells; Inhibition of colony formation; Increased cell volume, indicating cell differentiation	- ATRA and PB-4 do not induce differentiation of B16 cells and cause cell cycle arrest at the G0/G1 phase;<br>- lncRNA-Gm31932 may be involved in regulating cell cycle arrest and the differentiation of B16 cells induced by ATRA or PB-4;<br>- Silencing lncRNA-Gm31932 may induce differentiation of B16 cells;<br>- Silencing lncRNA-Gm31932 may induce cell cycle arrest at the G0/G1 phase;<br>- LncRNA-Gm31932 can bind to miR-344d-3-5p;<br>- LncRNA-Gm31932 acts as a sponge for miR-344d-3-5p, and its silencing may promote the expression of this miRNA;<br>- Prc1 and Nuf2 are direct target genes of miR-344d-3-5p;<br>- Prc1 and Nuf2 can cause cell cycle arrest;<br>- LncRNA-Gm31932 regulates the levels	The authors declare that they have no competing interests.	- A well-written article with a well-designed and clearly described experimental setup;<br>- It contains too much information for a single article; there is so much data and so many results that it becomes difficult to connect them all.
										Flow cytometry	Cell cycle phase	Increase in G0/G1-phase			
										Melanin content assay	Melanin production	Increased melanin			
										RNA-seq	Detection of	102 lncRNAs, 24 miRNAs,			
										RT-qPCR	Levels of lncRNAs	Validation of data for the 11			
											Expression of	Reduction after 24–72 hours			
										RNA FISH	Location of lncRNA-	lncRNA-Gm31932 is highly			
								Cell culture (B16 cells treated with ATRA or PB-4)	siRNA Transfection	Immunofluorescence	Biological function of lncRNA-Gm31932	From among the three siRNAs, they selected the one with the strongest inhibitory effect: siRNA-Gm31932-174			
									Transfection with siRNA-174	Microscopy		Decrease in cell density and			
										CCK8		Reduced colony formation			
										Melanin content assay		Melanin content and			
										Flow cytometry		A lower percentage of cells			
									Transfection with			Cell cycle arrest and			
						In silico		N/A		DIANA-LncBase and	Analysis of the	Mir-344d-3-5p can bind to			
						In vitro		Cell culture (B16 cells treated with ATRA or PB-4)	Co-transfection of wild type and mutant genes with luciferase and miR-344d-3-5p mimics and mutants	Luciferase	Binding of the	Mimetizante reduced			
										RNA pull-down	miR-344d-3-5p level	Higher than in the control			
										CCK8, microscopy,	Regulation of	LncRNA-Gm31932 can bind			
						In silico		N/A		Integrative analysis of	Target genes	Prc1 and Nuf2 are potential			
								Cell culture (B16	Co-transfection with	Dual-luciferase	Luciferase activity	Decreased luciferase activity			

						In vitro		[illegible] cells treated with ATRA or PB-4)	[illegible] miR-344d-3-5p mimic, Prc1-WT, and Nuf2-WT	WB	Prc1 and Nuf2 levels	Mir-344d-3-5p mimic	- LncRNA-Gm31932 regulates the levels of cycle-related proteins and molecules in the Wnt/β-catenin pathways via the miR-344d-3-5p/Prc1 and Nuf2 axis, inducing cell cycle arrest and differentiation;<br>- Silencing of lncRNA-Gm31932 may inhibit melanoma growth via the miR-344d-3-5p/Prc1 and Nuf2 axis.		
										RT-qPCR and WB	mRNA and protein	Lower levels			
								Cell culture (B16 cells)	Silencing of lncRNA-Gm31932	WB	The Prc1 and Nuf2	Inhibition of Prc1 and Nuf2			
											Expression levels of proteins related to the cell cycle	CDK2, CDC2, and cyclin B1 decreased following silencing of lncRNA-Gm31932, and the levels of p21 and p27 increased			
											WNT1 and β-catenin	Reduced levels due to the			
						In vivo	n = 24 (8 mice per group, 3 groups)	C57BL/6 male mice	Tumor xenograft with siRNA-Gm31932-174 insertion	Tumor volume and	The potential of	Volume and weight have			
										Immunohistochemical	Ki-67 expression	Ki-67 expression was			
										RT-qPCR	Levels of lncRNA	Levels of lncRNA-Gm31932			
										Melanin content assay	Melanin levels and	Tissues treated with siRNA-			
										RT-qPCR, WB, and	Prc1 and Nuf2 levels	In the tissues treated with			
										WB	Levels of CDK2,	Tissues treated with siRNA-			
											Wnt and β-catenin	In the tissues treated with			
[55] 10.1007 /s12032-016-0804-2	Yanping Wei, Qianqian Sun, Lindong Zhao, Jianbo Wu, Xiaonan Chen, Yuanyuan Wang, Wenqiao Zang, Guoqiang Zhao	2016	LncRNA UCA1-miR-507-FOXM1 axis is involved in cell proliferation, invasion and G0/G1 cell cycle arrest in melanoma	Medical Oncology (London) Impact Factor 3.5	Department of Dermatology, The People's Hospital of Jiaozuo City, Jiaozuo, 454000, Henan, China.	Ex vivo and in vitro	n = 57 (18 primary melanomas, 19 metastatic melanomas, 20 benign nevi controls)	Tissues from metastatic melanoma, primary melanoma, melanocytic nevi, and cell cultures (A375 and SK-MEL-2)	N/A	RT-PCR	Expression of the lncRNA UCA1 and FOXM1 mRNAs	Overexpression of UCA1 and FOXM1 in primary and metastatic melanoma and in A375 and SK-MEL-2	- UCA1 is overexpressed in melanoma tumor tissues, as well as in the A375 and SK-MEL-2 cell lines;<br>- Depletion of the lncRNA UCA1 can suppress cell proliferation and invasion and can also induce cell cycle arrest at the G0/G1 phase;<br>- MiR-507 can bind directly to UCA1 at the miRNA recognition site;<br>- There is a negative correlation between miR-507 and UCA1;<br>- FOXM1 is a target of miR-507 and can be downregulated by both miR-507 overexpression and UCA1 depletion;<br>- There is a UCA1-miR-507-FOXM1 interaction that can inhibit cell proliferation and invasion, as well as induce cell cycle arrest at the G0/G1 phase.	The authors declare no conflict of interest or financial disclosures of this study.	- The article is very easy to read and understand; it is quite straightforward;<br>- The graph in Figure 2 is poorly presented;<br>- Some captions are incorrect; for example, SK-MEL-2 is labeled as SK-DEL-2 in some graphs.
						In vitro	N/A	Cell culture (A375 and SK-MEL-2)	Transfection of scrambled UCA1 and siRNA targeting UCA1	RT-PCR and CCK-8	Confirm whether the	Cells expressing siRNA-			
										Transwell	Cell invasion	When UCA1 is depleted, the			
										Flow cytometry	Cell cycle	Cells treated with siRNA-			
						In silico		N/A		miRcode	Possible binding	miR-507 was selected			
						Ex vivo	n = 40 (20	Melanoma tissues	N/A	RT-qPCR	miR-507 and UCA1	An inversely proportional			
						In vitro	N/A	Cell culture (A375 and SK-MEL-2)	UCA1 siRNA Transfection	RT-qPCR	miR-507 expression	Increased expression of miR-507			
									Overexpression of		UCA1 expression	Inhibition of UCA1			
									UCA1 siRNA Transfection	Luciferase	Relationship between UCA1 and miR-507	miR-507 suppressed luciferase activity in wild-type UCA1 lncRNA, but did not affect that of the mutant			
									Antibody against Ago2	RIP	Mechanism	Both miR-507 and UCA1			
						In silico		N/A		TargetScan and	Targets of miR-507	Based on previous studies			
						In vitro		Cell culture (A375 and SK-MEL-2)	miR-507 mimic	Dual-luciferase	Verification of the	Confirmed			
									Transfection with si-UCA1, miR-507 mimic, and their combination	WB, qPCR	Relationship between FOXM1, UCA1, and miR-507	FOXM1 mRNA was reduced in the groups treated with si-UCA1, miR-507 mimics, and si-UCA1+miR-507 mimics; the same was true for its protein			
										CCK-8, Transwell, and	Cell behavior	Reduced UCA1 expression			
						In vitro and in silico	N/A	Primary tumor (Mel Ho and A375), metastasis (501 Mel and Lu 1205), healthy melanocytes	N/A	GEO	Expression of 28,536 genes involved	List of genes that are up- and down-regulated	- Overexpression of the miR-194-5p mimic leads to a reduction in HuR protein levels;<br>- Higher HuR expression in Mel Wei and much higher in Mel Im;		
						In silico					Transcripts	The number of these			
											HuR expression	Upregulated compared to			
											Selection of genes	150 ARE genes selected at			
						In vitro		Lysates from healthy cells, primary melanoma (Mel Juso, Mel Wei, and Mel Ho), and metastatic melanoma (SkMel28 and HTZ19)	N/A	WB	Analysis of HuR protein levels	Increased in all cell lines, particularly in metastatic ones			
						In silico		N/A		GEO	HuR mRNA levels	Upregulation of HuR mRNA			
						Ex vivo	Not specified		N/A	Immunohistochemistry		High levels of HuR mRNA expression, particularly in metastatic tissues			
						In silico	N/A			TCGA	Patient survival	Elevated HuR levels are			
										TargetScan	Screening of	miR-194-5p was identified in			
						In vitro	N/A	Cell culture (Mel Wei and Mel Im)	Transfection of the miR-194-5p mimic	Luciferase	HuR levels	Decreased luciferase activity in the HuR3'UTR			
								[illegible] lysates, primary		WB, qPCR	Location of HuR	HuR is localized to the			

[36] 10.3390 /cancers12051299	Janika K Liebig, Silke Kuphal, Anja Katrin Bosserhoff	2020	HuRdling Senescence: HuR Breaks BRAF-Induced Senescence in Melanocytes and Supports Melanoma Growth	Cancers Impact Factor: 4.4	Institute of Biochemistry, Emil-Fischer Zentrum, Friedrich-Alexander University of Erlangen-Nürnberg (FAU), 91054 Erlangen, Germany.			melanoma (Mel	Nuclear and cytoplasmic fractions	Immunofluorescence	Anti-HuR antibody	Low or absent levels in	- Expression and localization change as the disease progresses;<br>- Loss of HuR results in reduced cell proliferation by inducing a G1/G0 cell cycle arrest;<br>- Overexpression of HuR is capable of reversing the antiproliferative effect of BRAF OIS in NHEM cells;<br>- HuR functions as a potential stabilizer of MITF, influencing cell proliferation and the senescent phenotype;<br>- HuR can overcome senescence and drive the development of nevus-derived melanoma.	The authors declare that they have no conflicts of interest.	- They are working with tissues and referring to the study model as "in vivo," when the correct term would be "ex vivo";<br>- The search for microRNA was not clearly specified;<br>- There is a lack of information about the samples used throughout the article.
						Ex vivo	Not specified				Location of HuR	In primary melanoma tissue, HuR is found primarily in the nucleus and rarely in the cytoplasm, whereas in metastatic melanoma, it is found primarily in the cytoplasm			
						In vitro	N/A	Cell culture (Mel Wei and Mel Im)	siRNA transfection for HuR	xCELLigence (RTCA)	Effect of HuR knockdown on cell proliferation	Inhibitory effect on the growth of both cell lines			
										XTT Assay		Confirmation of previous			
										Clonogenic assay	The cell's ability to form colonies from isolated cells	After KD treatment, the			
												Only the knockout of HuR led			
										PI/Annexin staining	Apoptosis	No significant difference in			
										Flow cytometry	Cell cycle	Increases in Mel Wei and Mel Im cells transfected with siHuR in the G1/G0 phase, suggesting cell cycle arrest following HuR knockdown			
										Luciferase	Observation at the	Decreased activity of the AP-			
										WB	Cyclin D1	Reduced in melanoma cells			
										(SA)-β-galactosidase	Analysis of whether	Strong staining following			
										Immunofluorescence	Analysis of PML	Significant accumulation of			
											Levels of	Upregulation of histones			
										WB	MITF protein levels	Reduced in the presence of			
								Cell culture (OIS cells overexpressing HuR and healthy cells)	Overexpression of HuR	(SA)-β-galactosidase	Senescence	Reduced staining in BRAF-			
										Immunofluorescence	PML and H3K9	There was a reduction in			
											Ki-67 Proliferation	Overexpression of HuR can			
						Ex vivo	n = 80 (23 nevi and 57 nevus-derived melanomas)	Nevus and melanoma	N/A	RNA-seq	HuR expression	Increased in melanomas			
										WB	MITF mRNA expression	HuR OE upregulated MITF mRNA			
[56] 10.1038/onc.2012.324	A Bhattacharya, U Schmitz, O Wolkenhauer, M Schönherr, Y Raatz, M Kunz	2012	Regulation of cell cycle checkpoint kinase WEE1 by miR-195 in malignant melanoma	Oncogene Impact Factor 7.3	Department of Dermatology, University of Leipzig, Leipzig, Germany.	Ex vivo	n = 10 (formalin-fixed paraffin-embedded human tissue samples)	Tissues from primary melanoma and metastases	N/A	Immunohistochemistry	WEE1 expression	Metastatic cells have lower concentrations of WEE1 than those of primary melanoma	- There is an inverse correlation between WEE1 protein expression and the aggressiveness of melanoma cells;<br>- WEE1 is downregulated during melanoma progression, and tumor growth may depend on this downregulation;<br>- MiR-195 shows an inverse correlation with WEE1 expression and may contribute to its downregulation;<br>- MiR-195 is a regulator of WEE1 expression;<br>- MiR-195 inhibits WEE1 expression by directly targeting its 3'UTR;<br>- Role of WEE1 in mediating cell cycle arrest at the G2/M phase;<br>- WEE1 depletion may be beneficial for cells by allowing them to escape G2/M arrest and partially re-enter the cell cycle;<br>- Under conditions of DNA damage mediated by chemotherapeutic agents, miR-195 is capable of negatively regulating G2/M cell cycle arrest through negative modulation of WEE1;<br>- Colony-forming ability depends on WEE1 levels;<br>- WEE1 plays an important role in inducing G2/M cell cycle arrest;<br>- miR-195-mediated cell cycle arrest in the G2/M phases can be reversed by WEE1 overexpression;<br>- miR-195 increases melanoma cell migration independently of WEE1.	The authors declare no conflict of interest.	- The charts and images contain some errors;<br>- At the end of the article, several test results were simply listed, with no apparent order, reasoning, or explanation.
							n = 16 (8			RT-qPCR	WEE1 mRNA	WEE1 mRNA expression			
						In vitro	N/A	Cell cultures (non-		Immunoblotting	WEE1 expression	WEE1 expression was			
						In silico		N/A		TargetScan, PicTar,	miRNA targets	miR-195, miR-155, miR-372			
						Ex vivo	n = 16 (8	Tissues from		TaqMan miRNA	Screening of the	miR-195 is upregulated in			
						In silico	N/A			TargetScan and	The action of miR-	Highly conserved 3'UTR			
						In vitro	N/A	Cell culture (SK-MEL-28)	Transfection with miR-	RT-qPCR	WEE1 mRNA	Decreased 48 hours after			
									Transfection with miR-	Immunoblotting	Inhibitory activity of	WEE1 levels were reduced			
								Cell culture (SK-	A 3'UTR region clone	Luciferase	Site of WEE1	Overexpression of miR-195			
								Cell culture (SK-MEL-28)	Doxorubicin, vinblastine, or cisplatin	Flow cytometry	Cell cycle	The three agents induced			
										WB	Phosphorylation of	Increase along with the cycle			
									Non-targeted miR-195, siRNA for WEE1, miR-195, or antagomiR-195	Immunoblotting	WEE1 Mediation	Downregulation of WEE1			
										Flow cytometry	Effect of reduced phosphorylation at Cdc2-Y15	Cells expressing only miR-195 showed no difference. Cell cycle arrest decreased in the presence of siRNA targeting WEE1 in combination with miR-195, and the proportion of cells in the G0/G1 phase increased.			
									Transduction using a	RT-qPCR and	WEE1 expression	Higher WEE1 expression in			
								Cell culture (SK-MEL-28-WEE1 and SK-MEL-28)	N/A	CCK-8	Effects of WEE1	Decrease in the number of			
									Doxorubicin	Flow cytometry	Cell cycle	Higher percentage of cells stopping at the G2/M checkpoint in cells with WEE1			
									Doxorubicin + miR-195 transfection			A higher percentage of cells			
										WB	Expression of Cdc2-	Overexpression of WEE1 re-			
								Cell culture (SK-MEL-28)	Transfection of miR-195 and siRNA-WEE1	CCK-8 assay	Cell proliferation	Increased			
									AntagomiR-195			It decreased, but the change was not statistically significant			
									Transfection with miR-		Healing	It was faster			

								Reduced expression of WEE1	Wound healing assay	Migration capacity	No change			
								AntagomiR-195			Decreased			
[57] 10.1186 /s12885-018-4233-9	Yuxia Li, Jie Zhang, Yajing Liu, Bingyue Zhang, Fubo Zhong, Shubin Wang, Zhengyu Fang	2018	MiR-30a-5p confers cisplatin resistance by regulating IGF1R expression in melanoma cells	BMC Cancer Impact Factor 3.4	Biomedical Research Institute, Shenzhen Peking University-The Hong Kong University of Science and Technology Medical Center, No. 1120 Lianhua Road, Futian District, Shenzhen, Guangdong province, China	In vitro	N/A	Cell culture (M8/DDP and SK-Mel-19/DDP)	Continuous exposure to cisplatin for 5 months and 50 cell passages	MTS	Drug resistance index	Resistant strains have been successfully established	- There is an association between miR-30a-5p and cisplatin resistance in melanoma cells;<br>- miR-30a-5p cannot induce the degradation of IGF1R;<br>- There is a direct correlation between IGF1R and cisplatin resistance in melanoma cells;<br>- IGF1R may play a protective role against cisplatin-mediated DNA damage by regulating the cell cycle through the AKT/P53 pathway.	We declare that we have no financial or personal relationships with other individuals or organizations that could inappropriately influence our work. There is no professional or personal interest of any nature or kind in any product, service, and/or company that could be interpreted as influencing the position presented or the review of the manuscript titled "MiR-30a-5p confers cisplatin resistance by regulating IGF1R expression in melanoma cells."	The charts are cluttered and overly colorful, and they aren't standardized.
									RNA microarray, qPCR	Screening of	21 miRNAs identified			
									qPCR	Expression of miR-	Increased in the cells studied			
								Transfection of miR-30a-5p mimic and inhibitor	MTS	Cell resistance	The IC50 increased in M8			
											The IC50 decreased with the			
								Transfection of TuD-miR-30a-5p	qPCR	KD Efficiency	Transfection reduced the			
											The IC50 of the cells			
						In silico		N/A		microRNA.org, miRDB,	Identify potential	IGF1R has two putative			
						In vitro		Cell culture (M8/DDP and SK-Mel-19/DDP)	N/A	WB	IGF1R levels	Lower levels in both strains			
									Transfection of miR-			Reduction of IGF1R protein			
									Cloning of 3'UTR	Luciferase	Testing of the	When the wild-type was			
									Transfection with two types of siRNA	RT-PCR	Possible role of IGF1R in cellular resistance	miR-30a-5p expression			
												With the elimination of			
										WB	Expression of AKT	AKT phosphorylation was			
										Flow cytometry	Cell cycle	Cell cycle arrest, where a			
												The G2/M phase was			
[58] 10.1186 /s12967-024-05527-7	Vladimir Bezrookove, Imran Khan, Anukana Bhattacharjee, Juifang Fan, Robyn Jones, Anima Sharma, Mehdi Nosrati, Pierre-Yves Desprez, Nathan Salomonis, Yihui Shi, Altaf Dar, Mohammed Kashani-Sabet	2024	miR-876-3p is a tumor suppressor on 9p21 that is inactivated in melanoma and targets ERK	Journal of Translational Medicine Impact Factor: 7.5	California Pacific Medical Center (CPMC) Research Institute, 475 Brannan St., Suite 130, San Francisco, CA, 94107, USA	Ex vivo and in vitro	n = 103 (55 melanoma tissues and 48 nevi)	Benign nevi and melanoma tissue; normal melanocytes and melanoma melanocytes	N/A	RT-qPCR	miR-876 expression levels	Most samples showed downregulation of miR-876 in primary melanoma tissue; downregulation was also observed in melanoma cell lines	- The loss of miR-876 copy number, as well as its downregulated expression levels, support a tumor suppressor role;<br>- miR-876 expression is reduced in a substantial proportion of melanoma cell lines alongside CDKN2A expression;<br>- miR-876 regulates cell growth, migration, invasion, and apoptosis, a functional role as a tumor suppressor;<br>- ERK is a target of miR-876 action.	The authors declare that they have no competing interests.	- The results are presented in a continuous format, without headings;<br>- Most of the experiments do not specify which test was performed; they are not mentioned either in the text or in the captions of the figures and graphs. Therefore, it is necessary to correlate the methods described in the Materials and Methods section with the experiments conducted and the results presented;<br>- The reason for working with this microRNA was not explained.
						In vitro	N/A	Melanoma TCGA			Number of copies of	Deletions in 60% of cases			
								Cell culture (HEM, A375, LOX, Mamel12, 1205-LU, C8161.9)		RT-qPCR	CDKN2A expression level, copy number, and mutational status	Reduced expression of			
										FISH		Euploidy for CDKN2A in			
										NGS		Distinct point mutations in			
								Cell culture (C8161.9 and A375)	Overexpression of miR-876	CCK-8	Function of miR-876	Inhibition of the colony-			
										Flow cytometry	Cell cycle	Arrests by G1 and a decline			
											Apoptosis	Induction of apoptosis			
										Transwell migration	Ability to migrate	Prevention of migration and			
						In vivo and ex vivo	n = 16 (8 control miR and 8 miR-876)	Athymic BALB/c nu/nu nude mice	Subcutaneous injection of C8161.9 cells > The resulting tumors received injections of miR-876 and a control miR	Measurement	Tumor growth	Reduction in the total volume of tumors treated with miR-876			
						Ex vivo	n = 103 (55 melanoma tissues and 48 benign nevi controls)	Tumors C8161.9	miR-876 and control miR	RNA-seq	Transcriptomic changes	2,469 genes with reduced expression and 702 with increased expression			
										Analysis of genetic ontology, RT-qPCR		Increased tumor suppressor			
												Reduced expression of CA9,			
						In silico	N/A			TargetScan, miRanda	Possible targets of	MAPK1/ERK2 in the 3'UTR			
						In vitro	N/A	Cell culture (C8161.9 and A375)	Co-transfection of the ERK3'UTR together with miR-876	Luciferase	Regulation of miR-	Reduction of luciferase in			
										WB	ERK expression	Overexpression of miR-876			
									Transfection of	CCK-8	Colony formation	Increased ability to form			
								Cell culture (A375)	Different	WB	Downregulation of	Overexpression of miR-876			
[59] 10.1007 /s13277-016-5271-z	Christopher Alderman, Ayoub Sehlaoui, Zhaoyang Xiao, Yixin Yang	2016	MicroRNA-15a inhibits the growth and invasiveness of malignant melanoma and directly targets on CDCA4 gene	Tumour Biology: The journal of the International Society for Oncodevelopmental Biology and Medicine Impact factor not available	Department of Biological Sciences, Emporia State University, 1 Kellogg Circle, Emporia, KS, USA	In vitro	N/A	Cell culture (SK-MEL-28, WM1552C, A375, and B16-F10)	Transfection for 48 hours with isolated transfection reagent, miR-15a mimic, or siRNA	MTT	Effect of miR-15a on cell viability	Reduced viability across all strains	- MiR-15a halts cell cycle progression at the G1/G0 phase;<br>- MiR-15a inhibited migration and invasiveness;<br>- AKT3 and CDCA4 are regulated downstream of the target protein or may be direct targets of miR-15a;<br>- miR-15a directly targets CDCA4 and downregulates its expression.	The authors (Christopher Alderman, Ayoub Sehlaoui, Zhaoyang Xiao, Yixin Yang) of the manuscript entitled BMicroRNA-15a Inhibits the Growth and Invasiveness of Malignant Melanoma and Directly Targets on CDCA4 Gene^ have NO affiliations with or involvement in any organization or entity with any financial interest, or non-financial interest in the subject matter or materials discussed in this manuscript.	explaining why;<br>- They conducted a feasibility test and concluded that the miR inhibits melanoma cell growth, but more experiments were needed to support the hypothesis;<br>- They conducted a test for each hypothesis and have already drawn conclusions. Again, more experiments are needed;<br>- They state the title, describe a characteristic of melanoma, and proceed directly to the results; I don't think this is necessary—this is something that should be addressed in the discussion;<br>- The graphs are not standardized; they are colored and have closed grids, and the
						In vivo	n = 24 (3	C57BL/6 mice	B16-F10 cells	Measurement	Tumor size	MiR-15a slowed tumor			
						In vitro	N/A	Cell culture (SK-MEL-28)	Transfection with 100 nM of miR-15a	Flow cytometry	Cell cycle	An increase in cells in the			
										Transwell	Migration and	MiR-15a reduced the number			
						In silico		N/A		TargetScanTM	Targets of miR-15a	AKT3 and CDCA4 with			
						In vitro		Cell culture (SK-	miR-15a mimic and	Immunobbloting	Expression levels of	A decrease in the levels of			
								Cell culture (SK-MEL-28)	Co-transfection of miR-15a mimic + CDCA4 3′ UTR luciferase reporter plasmid (WT and MUT)	Luciferase	Direct target of miR-15a	MiR-15a reduced luciferase activity			
						Ex vivo	Not specified	Melanoma tissue	N/A	RT-qPCR	miR-425 expression	Lower levels			- It started with miR-425 without explaining why;
								Cell culture (A375,							
										MTT	Cell viability	MiR-425 inhibited cell			

[60] 10.1016/j. biopha. 2015.08.010	Pei Liu, Yaotian Hu, Ling Ma, Min Du, Lin Xia, Zhensheng Hu	2015	miR-425 inhibits melanoma metastasis through repression of PI3K-Akt pathway by targeting IGF-1	Biomedicine & Pharmacotherapy Impact Factor 7.5	Department of Plastic Surgery, Qilu Hospital of Shandong University, Jinan, Shandong, China	In vitro	N/A	Cell culture (A375 and SK-MEL-28)	Transfection with miR-145 mimics or control	Transwell	Migration and	Decrease in migration and	- Low expression of miR-425 may be related to its tumor-suppressing role;<br>- miR-425 is negatively associated with the IGF1 protein.	Conflict of interest: not stated in the article	explaining why;<br>- Some graphs were poorly presented;<br>- The results are presented quickly, to the point, and clearly;<br>- Excellent captions, very well described, facilitate understanding of the experiments;<br>- Several omissions of information, mainly in the in vivo and ex vivo experiments;<br>- Did not disclose anything regarding conflicts of interest.
										WB	PCNA, AP-1, and	Decrease			
											p27 levels	Increase			
										CCK-8	Survival skills	Greater ability to form			
										Flow cytometry	Cell cycle	An increase in cells in the			
						In vivo	No in vivo	NUDE mice	A375 cells	Measurement	Tumor size	MiR-425 inhibited tumor			
						In silico	N/A			TargetScan	Target gene	IGF1			
						In vitro	N/A	Cell culture (A375)	Transfection with miR-	Luciferase	Confirmation of	Lower activity in the wild-type			
									Transfection with miR-425	RT-qPCR	IGF1 mRNA expression	Reduced			
										ELISA	IGF-1 expression				
								Cell culture (A375	Transfection with miR-	WB	Function of miR-425	It can inhibit IGF1-activated			
						Ex vivo	Not specified	Melanoma tissue	N/A	RT-qPCR	IGF-1 mRNA levels	Increase			
[61] 10.1038 /s41417-021-00313-9	Lijun Wu, Ke Li, Wei Lin, Jianjiang Liu, Qiang Qi, Guoliang Shen, Weixin Chen, Wenjun He	2022	Long noncoding RNA LINC01291 promotes the aggressive properties of melanoma by functioning as a competing endogenous RNA for microRNA-625-5p and subsequently increasing IGF-1R expression	Cancer Gene Therapy Impact Factor 5.0	Department of Plastic and Aesthetic Surgery, The Second Affiliated Hospital of Soochow University, Jiangsu, China	In silico	N/A			TCGA	Expression of LINC01291	Higher expression in melanoma tissues	- LINC01291 acts as a pro-oncogenic RNA during the development and progression of melanoma;<br>- LINC01291 acts as a sponge for miR-625-5p;<br>- miR-625-5p plays an inhibitory role in melanoma progression;<br>- LINC, miR, and the protein are present in the same RNA-induced silencing complex;<br>- After acting as a sponge for miR, LINC increases IGF-1R expression;<br>- LINC01291 plays pro-oncogenic roles in melanoma by regulating the miR-625-5p/IGF-1R axis;<br>- Loss of LINC reduces the chemoresistance of melanoma cells to cisplatin by regulating the miR-625-5p/IGF-1 axis.	The authors declare that they have no competing interests.	- The graphs are not visually appealing;<br>- This is a very data-rich study, but a single image contains 13 grouped graphs, which makes the layout cluttered and difficult to read;<br>- There are so many experiments that they couldn't fit everything into a single article; at a certain point, they start reporting only the results of a series of experiments without specifying which ones or how they were conducted;<br>- The ex vivo stage is poorly described.
						Ex vivo	n = 41 pairs (melanoma tissue samples)	Human melanoma tissue	N/A	RT-qPCR					
						In silico	N/A			TCGA	Clinical significance	No results found			
						In vitro	N/A	Cell culture (A375, HT-144, SK-MEL-1, A2058)		RT-qPCR	Expression of LINC01291	Highest expression			
								Cell culture (A375 and HT-144)	Transfection with siRNA-LINC01291		Silenciar LINC01291	Confirmado			
										CCK-8	Colony formation	Reduced cell proliferation			
										Flow cytometry	Cell apoptosis	Increase			
											Cell cycle	Pause in the G0/G1 phases			
										Transwell	Migration and	Decreased			
						In silico		N/A	N/A	LncLocator	Distribution of LINC01291	Found in the cytoplasm and nucleus, but mainly in the cytoplasm			
						In vitro		Cell culture (A375		Subcellular		Cytoplasm			
						In silico		N/A		ENCORI	miRNA targeted by	9 miRNAs identified			
										TCGA	Expression of the identified miRNAs	MiR-625-5p and miR-766-5p were downregulated in melanoma tissues			
						In vitro		Cell culture (A375 and HT-144)	Transfection with	RT-qPCR	Expression of miR-	MiR-625-5p is upregulated			
									Incubation with Ago2	RIP	Expression of miR-	Increased			
									Transfection of miR-	Luciferase	The link between	Luciferase activity is inhibited			
						In silico		N/A		TCGA	Regulation of miR-	Its low expression is			
						In vitro		Cell culture (A375 and HT-144)	Transfection with miR-625-5p mimic and control	RT-qPCR	Expression of miR-	It has increased			
										CCK-8	Colony formation	Inhibition of proliferation and			
										Flow cytometry	Cell apoptosis	Increase			
											Cell cycle	Pause in the G0/G1 phases			
										Transwell	Invasion and	Reduced			
						In silico		N/A		TargetScan	Target of miR-625-	IGF-1R			
						In vitro		Cell culture (A375	Mutant and wild-type	Luciferase	Possible connection	Luciferase activity was			
						Ex vivo	n = 41 pairs (melanoma tissue samples)	Human melanoma tissue	N/A	RT-qPCR	IGF-1R expression	Highly expressed and inversely associated with LINC expression			
						In vitro	N/A	Cell culture (A375 and HT-144)		RT-qPCR and WB	Regulatory effects of	Upregulation of miR reduced			
									LINC siRNA		IGF-1R expression	Decrease in IGF-1R mRNA			
									Ago2 and IgG	RIP	Regulation of LINC,	Upregulation			
									Co-transfection of anti-miR-625-5p or anti-NC + siLINC	RT-qPCR and WB	IGF-1R expression	mRNA and IGF-1R protein			
										CCK-8	Colony formation	Reduced proliferation and			
									Inhibition of miR-625-5p			Back to normal			
										Flow cytometry	Cell apoptosis	Balance of apoptosis			
											Cell cycle	Pause in the G0/G1 phases			
										Transwell	Migration and invasion	Inhibited by LINC loss			
									Co-transfection of anti-			Reversed inhibition effect			
									Co-transfection of siLINC + IGF-1R plasmid and pc-IGF-1R	WB	Transfection	Confirmed			
										RT-qPCR, CCK-8,	Effects of siRNA-	IGF-1R reversed the			
								Cell culture (A375)	sh-LINC	RT-qPCR	Impact of sh-LINC	Reduces LINC expression			
						In vivo	n = 6 (3 sh-LINC01291 and 3 sh-NC)	BALB/c nude mice	Subcutaneous injection of A375 cells with LINC or a control	RT-qPCR	Cotransfection efficiency	Confirmed			
										Measurement	Tumor volume and	Decreased			
						Ex vivo	n = 6 tumor xenografts (derived from 6 mice)	Tumor xenograft	N/A	RT-qPCR	Expression levels of LINC, miR, and IGF-1R	Decreased LINC and IGF-1R, increased miR when LINC is decreased			

							6 mice)		siLINC	WB	Levels of IGF-1R,	Decreased levels and			
						In vitro	N/A	Cell culture (LINC-silenced A375 and HT-144 cells)	Cisplatin	CCK-8	Cell viability	Low survival rate following cisplatin treatment			
[50] 10.1186 /s13046-024-03129-1	Adriana Rosa Gambardella, Caterina Antonucci, Cristiana Zanetti, Francesco Noto, Sara Andreone, Davide Vacca, Valentina Pellerito, Chiara Sicignano, Giuseppe Parrottino, Valentina Tirelli, Antonella Tinari, Mario Falchi, Adele De Ninno, Luca Businaro, Stefania Loffredo, Gilda Varricchi, Claudio Tripodo, Claudia Afferni, Isabella Parolini, Fabrizio Mattei, Giovanna Schiavoni	2024	IL-33 stimulates the anticancer activities of eosinophils through extracellular vesicle-driven reprogramming of tumor cells	Journal of Experimental & Clinical Cancer Research: CR Impact Factor 12.8	Department of Oncology and Molecular Medicine, Istituto Superiore Di Sanità, Rome, Italy	In vitro	N/A	Cell culture (mouse Eo33 and Eo5)	N/A	Ultracentrifugation and NTA	Isolation and characterization of EVs	Vesicle population size and exclusion of the presence of lytic granules	- Activation with IL-33 stimulates the secretion of EVs by eosinophils, which also occurs in the vicinity of tumor cells;<br>- Both Eo5-EV and Eo33-EV are efficiently taken up by tumor cells;<br>- EVs from IL-33-activated eosinophils inhibit tumor growth by blocking the cell cycle;<br>- EVs from IL-33-activated eosinophils affect the gene program related to epithelial-to-mesenchymal transition (EMT) in target tumor cells, reshaping their phenotype to one with lower metastatic potential;<br>- IL-33 activation of eosinophils affects the phenotype of human cells as well.	The authors declare that they have no competing interests.	- A very good article—thorough and comprehensive;<br>- The only thing missing was a more detailed specification of the sample size for the in vivo experiments.
										MET	Ultrastructure and	N/A			
										WB	Tsg101 and Alix	Absence of contaminating			
									Bodipy FL C16	Flow cytometry	EV Rollout	It released more EVs			
								Cell culture (Eo33 cells labeled with FL C16)	Co-cultured with B16-F10	MET		Presence of multivesicular			
								Cell culture (B16-F10, MCA205, and TC1)	Co-culture of Eo5 and Eo33 labeled with FL C16	Transwell	Fluorescent EV	N/A			
										Flow cytometry	Incorporation of EVs	It happened quickly on B16			
								Cell culture (Eo labeled with FL C16 and tumor cells on a microfluidic chip)	Matrigel with separate lateral chambers connected by two microchannel arrays and a central fluidic chamber for the passage of EVs	Fluorescence microscopy	Fluorescence of EVs	After 18 hours, B16 exhibited green fluorescence from both types of Eo at the same intensity			
										Time-lapse microscopy	Green fluorescence	Efficient onboarding in 5			
										DAPI, confocal	Incorporation of EVs	In the cytoplasm of B16 cells			
								Cell culture (Eo	GW4869	Flow cytometry	Cell apoptosis	Eo33 required high doses to			
								Cell culture (B16-F10, MCA205, and TC1)	Eo33-EV	MTS	Cell proliferation	Reduced			
										Violet crystal	Cell coverage area				
										3D Culture	Spheroid formation	Increased			
										RNA-seq and RT-	Gene analysis	Elevated CDKI (Cdkn1B and			
										Flow cytometry	Cell cycle	Increase in the G0/G1 phase			
								Cell culture (Eo and B16-F10)	N/A	RT-qPCR and WB	Adjustment of the	Upregulation of E-			
										Flow cytometry	Cadherin expression	Found in the cell membrane			
										Scratch	Invasion	The untreated wounds			
										Morphometric analysis		Increased elongation and			
						In vivo	Not specified	C57BL/6 mice	Intravenous injection of	Count	Number of	A small number of lung			
						In vitro	N/A	Cell culture (Eo33 and Eo5 from mice)	N/A	RNA-seq	Molecular content of	149,222 transcripts, of which			
										Cross-filtration	Obtain the lncRNA and mRNA transcripts	11 lncRNAs upregulated in			
												88 mRNA upregulation in			
						In silico		N/A		Heat map	Data analysis	Similarity between two			
										CancerMine and		A higher number of tumor			
						In vitro		Eosinophils from healthy patients and A375P patients	Stimulation with IL-5 or IL-33	RT-qPCR	Expression analysis	Reduction in tumor growth			
										WB		Elevated levels of CDH1 and			
												Increased expression of			
