# Supplementary material for: MicroRNAs and Cellular Senescence in Melanoma: An Underexplored Link to Tumor Progression—A Systematic Review with Bioinformatics Analyses
Source: Int J Mol Sci. 2026 Jul 21;27(14):6462. doi: 10.3390/ijms27146462 (PMC13410509; doi:10.3390/ijms27146462)
Supplement: Supplementary file 1 [file ijms-27-06462-s001.zip › Table S2 - Supplementary Material.xlsx - Planilha1.pdf]

Article: [51] 10.1016/j.jid.2017.03.039		
In vitro / Ex vivo		
**Assessment criterion**	**Evaluation question**	**Points**
Experimental control	Is there an appropriate control group?	1
Replication	Are there enough biological/technical replicates?	1
Standardization	Are the methods well-described and reproducible?	1
Cellular identity	Has the cell line been authenticated?	0
Contamination	Have you reported a mycoplasma test or contamination?	0
Experimental conditions	Are the conditions (time, dose, medium) clearly defined?	1
Blinding	Was the evaluation of the results blinded? (if applicable)	N/A
Statistical analysis	Is the analysis appropriate and well-described?	1
In vivo		
**Assessment criterion**	**Evaluation question**	**Points**
Randomization	Were the animals randomized?	N/A
Hiding allocation	Was the group selected without bias?	N/A
Initial similarity	Were the groups similar at the beginning?	N/A
Blinding (procedure)	Was the researcher unaware of the treatment?	N/A
Blinding (assessment)	Was the person evaluating the results blind?	N/A
Incomplete data	Have the losses of animals been explained?	N/A
Experimental conditions	Standardized environment and management?	N/A
**Score**		**0.71**
**Article: [52] 10.1097/MD.0000000000001327**		
**In vitro / Ex vivo**		
**Assessment criterion**	**Evaluation question**	**Points**
Experimental control	Is there an appropriate control group?	1
Replication	Are there enough biological/technical replicates?	1
Standardization	Are the methods well-described and reproducible?	0
Cellular identity	Has the cell line been authenticated?	0
Contamination	Have you reported a mycoplasma test or contamination?	0
Experimental conditions	Are the conditions (time, dose, medium) clearly defined?	0
Blinding	Was the evaluation of the results blinded? (if applicable)	N/A
Statistical analysis	Is the analysis appropriate and well-described?	1
**In vivo**		
**Assessment criterion**	**Evaluation question**	**Points**
Randomization	Were the animals randomized?	1
Hiding allocation	Was the group selected without bias?	1
Initial similarity	Were the groups similar at the beginning?	0
Blinding (procedure)	Was the researcher unaware of the treatment?	0
Blinding (assessment)	Was the person evaluating the results blind?	0
Incomplete data	Have the losses of animals been explained?	0
Experimental conditions	Standardized environment and management?	0
**Score**		**0.36**
**Article: [53] PMC5250706**		
**In vitro / Ex vivo**		
**Assessment criterion**	**Evaluation question**	**Points**

Experimental control	Is there an appropriate control group?	0
Replication	Are there enough biological/technical replicates?	1
Standardization	Are the methods well-described and reproducible?	0
Cellular identity	Has the cell line been authenticated?	0
Contamination	Have you reported a mycoplasma test or contamination?	0
Experimental conditions	Are the conditions (time, dose, medium) clearly defined?	0
Blinding	Was the evaluation of the results blinded? (if applicable)	N/A
Statistical analysis	Is the analysis appropriate and well-described?	1
**In vivo**		
**Assessment criterion**	**Evaluation question**	**Points**
Randomization	Were the animals randomized?	0
Hiding allocation	Was the group selected without bias?	0
Initial similarity	Were the groups similar at the beginning?	0
Blinding (procedure)	Was the researcher unaware of the treatment?	0
Blinding (assessment)	Was the person evaluating the results blind?	0
Incomplete data	Have the losses of animals been explained?	0
Experimental conditions	Standardized environment and management?	1
**Score**		**0.21**
**Article: [54] 10.1186/1471-5945-14-8**		
**In vitro / Ex vivo**		
**Assessment criterion**	**Evaluation question**	**Points**
Experimental control	Is there an appropriate control group?	0
Replication	Are there enough biological/technical replicates?	1
Standardization	Are the methods well-described and reproducible?	1
Cellular identity	Has the cell line been authenticated?	0
Contamination	Have you reported a mycoplasma test or contamination?	0
Experimental conditions	Are the conditions (time, dose, medium) clearly defined?	1
Blinding	Was the evaluation of the results blinded? (if applicable)	1
Statistical analysis	Is the analysis appropriate and well-described?	1
**In vivo**		
**Assessment criterion**	**Evaluation question**	**Points**
Randomization	Were the animals randomized?	0
Hiding allocation	Was the group selected without bias?	0
Initial similarity	Were the groups similar at the beginning?	1
Blinding (procedure)	Was the researcher unaware of the treatment?	0
Blinding (assessment)	Was the person evaluating the results blind?	0
Incomplete data	Have the losses of animals been explained?	0
Experimental conditions	Standardized environment and management?	1
**Score**		**0.47**
**Article: [31] 10.3892/or.2018.6633**		
**In vitro / Ex vivo**		
**Assessment criterion**	**Evaluation question**	**Points**
Experimental control	Is there an appropriate control group?	1
Replication	Are there enough biological/technical replicates?	1

Standardization	Are the methods well-described and reproducible?	1
Cellular identity	Has the cell line been authenticated?	0
Contamination	Have you reported a mycoplasma test or contamination?	1
Experimental conditions	Are the conditions (time, dose, medium) clearly defined?	1
Blinding	Was the evaluation of the results blinded? (if applicable)	N/A
Statistical analysis	Is the analysis appropriate and well-described?	1
**In vivo**		
**Assessment criterion**	**Evaluation question**	**Points**
Randomization	Were the animals randomized?	0
Hiding allocation	Was the group selected without bias?	0
Initial similarity	Were the groups similar at the beginning?	1
Blinding (procedure)	Was the researcher unaware of the treatment?	0
Blinding (assessment)	Was the person evaluating the results blind?	0
Incomplete data	Have the losses of animals been explained?	0
Experimental conditions	Standardized environment and management?	1
**Score**		**0.57**
**Article: [30] 10.1038/s41419-022-04736-6**		
**In vitro / Ex vivo**		
**Assessment criterion**	**Evaluation question**	**Points**
Experimental control	Is there an appropriate control group?	1
Replication	Are there enough biological/technical replicates?	1
Standardization	Are the methods well-described and reproducible?	1
Cellular identity	Has the cell line been authenticated?	1
Contamination	Have you reported a mycoplasma test or contamination?	0
Experimental conditions	Are the conditions (time, dose, medium) clearly defined?	1
Blinding	Was the evaluation of the results blinded? (if applicable)	N/A
Statistical analysis	Is the analysis appropriate and well-described?	1
**In vivo**		
**Assessment criterion**	**Evaluation question**	**Points**
Randomization	Were the animals randomized?	1
Hiding allocation	Was the group selected without bias?	1
Initial similarity	Were the groups similar at the beginning?	1
Blinding (procedure)	Was the researcher unaware of the treatment?	0
Blinding (assessment)	Was the person evaluating the results blind?	0
Incomplete data	Have the losses of animals been explained?	0
Experimental conditions	Standardized environment and management?	1
**Score**		**0.71**
**Article: [55] 10.1007/s12032-016-0804-2**		
**In vitro / Ex vivo**		
**Assessment criterion**	**Evaluation question**	**Points**
Experimental control	Is there an appropriate control group?	1
Replication	Are there enough biological/technical replicates?	1
Standardization	Are the methods well-described and reproducible?	1
Cellular identity	Has the cell line been authenticated?	0

Contamination	Have you reported a mycoplasma test or contamination?	0
Experimental conditions	Are the conditions (time, dose, medium) clearly defined?	1
Blinding	Was the evaluation of the results blinded? (if applicable)	N/A
Statistical analysis	Is the analysis appropriate and well-described?	1
**In vivo**		
**Assessment criterion**	**Evaluation question**	**Points**
Randomization	Were the animals randomized?	N/A
Hiding allocation	Was the group selected without bias?	N/A
Initial similarity	Were the groups similar at the beginning?	N/A
Blinding (procedure)	Was the researcher unaware of the treatment?	N/A
Blinding (assessment)	Was the person evaluating the results blind?	N/A
Incomplete data	Have the losses of animals been explained?	N/A
Experimental conditions	Standardized environment and management?	N/A
**Score**		**0.71**

**Article: [36] 10.3390/cancers12051299**		
**In vitro / Ex vivo**		
**Assessment criterion**	**Evaluation question**	**Points**
Experimental control	Is there an appropriate control group?	1
Replication	Are there enough biological/technical replicates?	1
Standardization	Are the methods well-described and reproducible?	0
Cellular identity	Has the cell line been authenticated?	0
Contamination	Have you reported a mycoplasma test or contamination?	0
Experimental conditions	Are the conditions (time, dose, medium) clearly defined?	1
Blinding	Was the evaluation of the results blinded? (if applicable)	1
Statistical analysis	Is the analysis appropriate and well-described?	1
**In vivo**		
**Assessment criterion**	**Evaluation question**	**Points**
Randomization	Were the animals randomized?	N/A
Hiding allocation	Was the group selected without bias?	N/A
Initial similarity	Were the groups similar at the beginning?	N/A
Blinding (procedure)	Was the researcher unaware of the treatment?	N/A
Blinding (assessment)	Was the person evaluating the results blind?	N/A
Incomplete data	Have the losses of animals been explained?	N/A
Experimental conditions	Standardized environment and management?	N/A
**Score**		**0.62**

**Article: [56] 10.1038/onc.2012.324**		
**In vitro / Ex vivo**		
**Assessment criterion**	**Evaluation question**	**Points**
Experimental control	Is there an appropriate control group?	0
Replication	Are there enough biological/technical replicates?	1
Standardization	Are the methods well-described and reproducible?	0
Cellular identity	Has the cell line been authenticated?	0
Contamination	Have you reported a mycoplasma test or contamination?	0
Experimental conditions	Are the conditions (time, dose, medium) clearly defined?	1

Blinding	Was the evaluation of the results blinded? (if applicable)	N/A
Statistical analysis	Is the analysis appropriate and well-described?	1
**In vivo**		
**Assessment criterion**	**Evaluation question**	**Points**
Randomization	Were the animals randomized?	N/A
Hiding allocation	Was the group selected without bias?	N/A
Initial similarity	Were the groups similar at the beginning?	N/A
Blinding (procedure)	Was the researcher unaware of the treatment?	N/A
Blinding (assessment)	Was the person evaluating the results blind?	N/A
Incomplete data	Have the losses of animals been explained?	N/A
Experimental conditions	Standardized environment and management?	N/A
**Score**		**0.43**
**Article: [57] 10.1186/s12885-018-4233-9**		
**In vitro / Ex vivo**		
**Assessment criterion**	**Evaluation question**	**Points**
Experimental control	Is there an appropriate control group?	1
Replication	Are there enough biological/technical replicates?	1
Standardization	Are the methods well-described and reproducible?	1
Cellular identity	Has the cell line been authenticated?	0
Contamination	Have you reported a mycoplasma test or contamination?	0
Experimental conditions	Are the conditions (time, dose, medium) clearly defined?	1
Blinding	Was the evaluation of the results blinded? (if applicable)	N/A
Statistical analysis	Is the analysis appropriate and well-described?	1
**In vivo**		
**Assessment criterion**	**Evaluation question**	**Points**
Randomization	Were the animals randomized?	N/A
Hiding allocation	Was the group selected without bias?	N/A
Initial similarity	Were the groups similar at the beginning?	N/A
Blinding (procedure)	Was the researcher unaware of the treatment?	N/A
Blinding (assessment)	Was the person evaluating the results blind?	N/A
Incomplete data	Have the losses of animals been explained?	N/A
Experimental conditions	Standardized environment and management?	N/A
**Score**		**0.71**
**Article: [58] 10.1186/s12967-024-05527-7**		
**In vitro / Ex vivo**		
**Assessment criterion**	**Evaluation question**	**Points**
Experimental control	Is there an appropriate control group?	1
Replication	Are there enough biological/technical replicates?	1
Standardization	Are the methods well-described and reproducible?	0
Cellular identity	Has the cell line been authenticated?	0
Contamination	Have you reported a mycoplasma test or contamination?	1
Experimental conditions	Are the conditions (time, dose, medium) clearly defined?	1
Blinding	Was the evaluation of the results blinded? (if applicable)	N/A
Statistical analysis	Is the analysis appropriate and well-described?	1

In vivo		
**Assessment criterion**	**Evaluation question**	**Points**
Randomization	Were the animals randomized?	1
Hiding allocation	Was the group selected without bias?	1
Initial similarity	Were the groups similar at the beginning?	0
Blinding (procedure)	Was the researcher unaware of the treatment?	0
Blinding (assessment)	Was the person evaluating the results blind?	1
Incomplete data	Have the losses of animals been explained?	1
Experimental conditions	Standardized environment and management?	1
**Score**		**0.71**
**Article: [59] 10.1007/s13277-016-5271-z**		
**In vitro / Ex vivo**		
**Assessment criterion**	**Evaluation question**	**Points**
Experimental control	Is there an appropriate control group?	0
Replication	Are there enough biological/technical replicates?	1
Standardization	Are the methods well-described and reproducible?	1
Cellular identity	Has the cell line been authenticated?	0
Contamination	Have you reported a mycoplasma test or contamination?	0
Experimental conditions	Are the conditions (time, dose, medium) clearly defined?	1
Blinding	Was the evaluation of the results blinded? (if applicable)	N/A
Statistical analysis	Is the analysis appropriate and well-described?	1
**In vivo**		
**Assessment criterion**	**Evaluation question**	**Points**
Randomization	Were the animals randomized?	1
Hiding allocation	Was the group selected without bias?	0
Initial similarity	Were the groups similar at the beginning?	1
Blinding (procedure)	Was the researcher unaware of the treatment?	0
Blinding (assessment)	Was the person evaluating the results blind?	0
Incomplete data	Have the losses of animals been explained?	0
Experimental conditions	Standardized environment and management?	1
**Score**		**0.5**
**Article: [60] 10.1016/j.biopha.2015.08.010**		
**In vitro / Ex vivo**		
**Assessment criterion**	**Evaluation question**	**Points**
Experimental control	Is there an appropriate control group?	1
Replication	Are there enough biological/technical replicates?	1
Standardization	Are the methods well-described and reproducible?	1
Cellular identity	Has the cell line been authenticated?	0
Contamination	Have you reported a mycoplasma test or contamination?	0
Experimental conditions	Are the conditions (time, dose, medium) clearly defined?	1
Blinding	Was the evaluation of the results blinded? (if applicable)	N/A
Statistical analysis	Is the analysis appropriate and well-described?	1
**In vivo**		
**Assessment criterion**	**Evaluation question**	**Points**

Randomization	Were the animals randomized?	N/A
Hiding allocation	Was the group selected without bias?	N/A
Initial similarity	Were the groups similar at the beginning?	N/A
Blinding (procedure)	Was the researcher unaware of the treatment?	N/A
Blinding (assessment)	Was the person evaluating the results blind?	N/A
Incomplete data	Have the losses of animals been explained?	N/A
Experimental conditions	Standardized environment and management?	N/A
**Score**		**0.71**

**Article: [61] 10.1038/s41417-021-00313-9**		
**In vitro / Ex vivo**		
**Assessment criterion**	**Evaluation question**	**Points**
Experimental control	Is there an appropriate control group?	1
Replication	Are there enough biological/technical replicates?	1
Standardization	Are the methods well-described and reproducible?	0
Cellular identity	Has the cell line been authenticated?	1
Contamination	Have you reported a mycoplasma test or contamination?	1
Experimental conditions	Are the conditions (time, dose, medium) clearly defined?	1
Blinding	Was the evaluation of the results blinded? (if applicable)	1
Statistical analysis	Is the analysis appropriate and well-described?	1
**In vivo**		
**Assessment criterion**	**Evaluation question**	**Points**
Randomization	Were the animals randomized?	1
Hiding allocation	Was the group selected without bias?	1
Initial similarity	Were the groups similar at the beginning?	1
Blinding (procedure)	Was the researcher unaware of the treatment?	0
Blinding (assessment)	Was the person evaluating the results blind?	0
Incomplete data	Have the losses of animals been explained?	1
Experimental conditions	Standardized environment and management?	1
**Score**		**0.8**

**Article: [50] 10.1186/s13046-024-03129-1**		
**In vitro / Ex vivo**		
**Assessment criterion**	**Evaluation question**	**Points**
Experimental control	Is there an appropriate control group?	1
Replication	Are there enough biological/technical replicates?	1
Standardization	Are the methods well-described and reproducible?	1
Cellular identity	Has the cell line been authenticated?	0
Contamination	Have you reported a mycoplasma test or contamination?	1
Experimental conditions	Are the conditions (time, dose, medium) clearly defined?	1
Blinding	Was the evaluation of the results blinded? (if applicable)	N/A
Statistical analysis	Is the analysis appropriate and well-described?	1
**In vivo**		
**Assessment criterion**	**Evaluation question**	**Points**
Randomization	Were the animals randomized?	0
Hiding allocation	Was the group selected without bias?	0

Initial similarity	Were the groups similar at the beginning?	0
Blinding (procedure)	Was the researcher unaware of the treatment?	0
Blinding (assessment)	Was the person evaluating the results blind?	0
Incomplete data	Have the losses of animals been explained?	0
Experimental conditions	Standardized environment and management?	1
**Score**		**0.5**
